# Targeting molecular quantum memory with embedded error correction†

**DOI:** 10.1039/d1sc01506k

**Published:** 2021-06-02

**Authors:** Selena J. Lockyer, Alessandro Chiesa, Grigore A. Timco, Eric J. L. McInnes, Tom S. Bennett, Inigo J. Vitorica-Yrezebal, Stefano Carretta, Richard E. P. Winpenny

**Affiliations:** Department of Chemistry and Photon Science Institute, The University of Manchester Oxford Road Manchester M13 9PL UK richard.winpenny@manchester.ac.uk; Università di Parma, Dipartimento di Scienze Matematiche Fisiche e Informatiche I-43124 Parma Italy; UdR Parma, INSTM I-43124 Parma Italy

## Abstract

The implementation of a quantum computer requires both to protect information from environmental noise and to implement quantum operations efficiently. Achieving this by a fully fault-tolerant platform, in which quantum gates are implemented within quantum-error corrected units, poses stringent requirements on the coherence and control of such hardware. A more feasible architecture could consist of connected memories, that support error-correction by enhancing coherence, and processing units, that ensure fast manipulations. We present here a supramolecular {Cr_7_Ni}–Cu system which could form the elementary unit of this platform, where the electronic spin 1/2 of {Cr_7_Ni} provides the processor and the naturally isolated nuclear spin 3/2 of the Cu ion is used to encode a logical unit with embedded quantum error-correction. We demonstrate by realistic simulations that microwave pulses allow us to rapidly implement gates on the processor and to swap information between the processor and the quantum memory. By combining the storage into the Cu nuclear spin with quantum error correction, information can be protected for times much longer than the processor coherence.

## Abstract 2

The implementation of a quantum computer requires protecting of information from noise and the ability to perform quantum gates. We present a molecular architecture providing both these ingredients, *via* an electronic spin 1/2 processor and a nuclear spin 3/2 memory.

## Graphical abstract

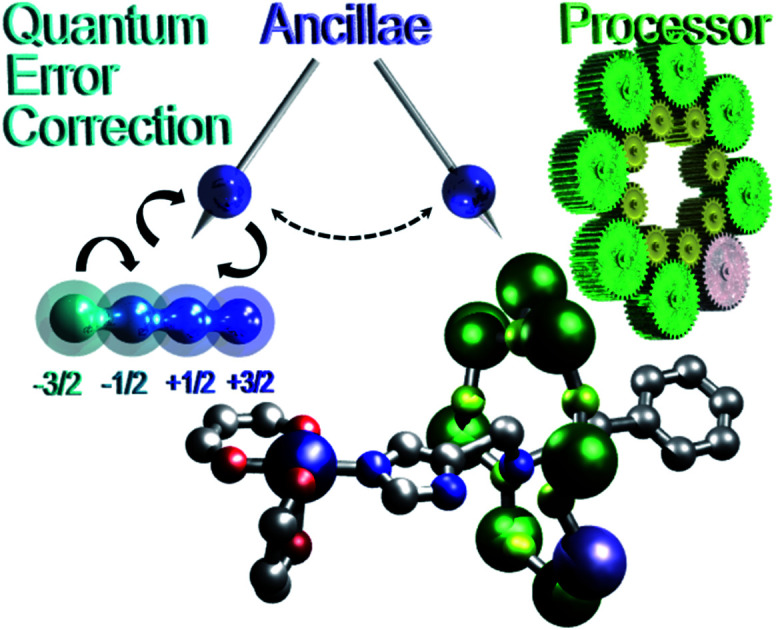

## Introduction

Since the original proposal by Leuenberger and Loss,^1^ there has been great interest in using electron spins in molecules for quantum information processing.^2–15^ The ability to modify molecules selectively means chemical systems offer advantages unavailable to solid state systems and has, for example, led to molecules showing long coherence times even to high temperatures.^6–23^ Another major advantage is the ability to bring together distinct electron spin qubits and potentially perform two qubit gates.^2,4,6,10–12,15^

Two further opportunities presented by molecules use nuclear spins. The first would be to store quantum information during the idle periods of complex quantum algorithms. During these idle periods in an algorithm, quantum information has to be stored in robust quantum memories that are isolated from the environment and protected from errors. This requires memory units characterized by coherence times much longer than those of the processors. A possibility is to use nuclear spins which are naturally more isolated than electron spins and hence less susceptible to decoherence due to noise.^24^ Therefore in a molecule you would need a processor, which uses electron spin for fast operations, and a nuclear spin, which would acts as a memory.

A further challenge in producing any scalable quantum computing platform is quantum error correction (QEC),^25–27^ which is needed to perform complex algorithms with high fidelity.^28^ Again molecules could offer a significant advantage. In classical computation error correction can use redundancy; information is stored multiple times and then the expectation is that not all “bits” will lose information simultaneously and thus errors are prevented. This is not possible in a quantum information processor.

The standard approach to QEC codes is due to Shor,^29^ and spreads the quantum information into an entangled state involving multiple physical qubits, *i.e.* multiple two-level systems such as *s* = 1/2 superconducting qubits. Molecules offer another possible opportunity for QEC as a single molecule could contain many entangled levels in one object if the nuclear spin quantum number *I* >1. There are many molecules where this could be possible, for example copper has *I* = 3/2 in both abundant isotopes. This is potentially a transformative advantage.

**Fig. 1:**
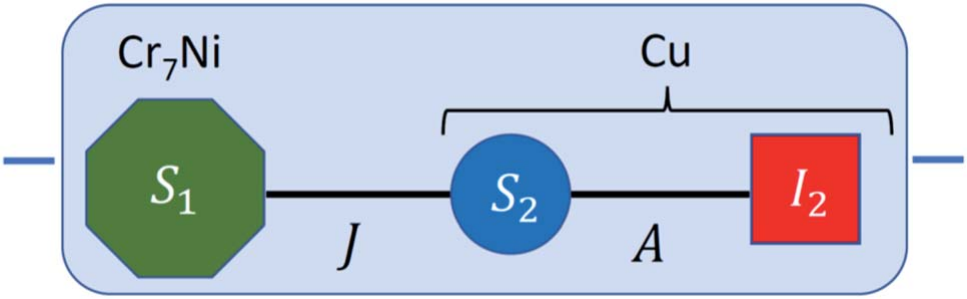
Scheme of the proposed quantum computing platform. Each elementary unit (blue shaded box) consists of a Cr_7_Ni qubit *S*_1_ = 1/2 (green octagon), used to process quantum information, coupled *via* an exchange interaction *J* to a Cu^II^ ion. The nuclear spin *I*_2_ = 3/2 provides a quantum memory, while the electronic Cu^II^ spin *S*_2_ = 1/2 (coupled to *I*_2_ by hyperfine interaction *A*) is exploited as an ancilla to perform quantum error correction cycles and to swap information from the nuclear memory to the electronic Cr_7_Ni processor.

Here we describe a supramolecular system that contain both an electron-spin based processor and an isolated, error-protected nuclear memory with very long coherence, along with the ability to swap information between the two sub-units (Fig. 1). A quantum hardware based on such a platform would allow us to separately (i) *process* quantum information by implementing quantum gates on the processors; (ii) rapidly *swap* information from the processor to the nuclear spin which acts as the quantum memory; (iii) *protect* quantum information by QEC cycles on the memories. By decoupling the memories from the processing units, we can efficiently implement quantum gates on part of the register, while keeping information stored in the memories protected for times much longer than the processor coherence^30,31^

In this work, we show that a {Cr_7_Ni}–Cu^II^ assembly is a very promising elementary unit for such a platform. In particular, {Cr_7_Ni} ring is used as the quantum processor, while the nuclear Cu spin 3/2 serves as a memory with embedded quantum error correction. Previously, {Cr_7_Ni} rings have demonstrated promising phase memory times^17^ and they can be organized into scalable architectures in which quantum operations can be implemented.^10–12^ Being based on electronic spins, {Cr_7_Ni} qubits can be easily manipulated by fast microwave pulses, allowing one to implement many gates before coherence is lost.

Importantly, we show here that quantum information can be swapped between memory and processing qubit very quickly. This is a fundamental step which may be repeated several times during a complex algorithm and hence needs to be implemented in times much shorter than the processor phase memory time. We demonstrate that this goal can be achieved by exploiting the effective interaction between the electronic {Cr_7_Ni} spin *S*_1_ and the nuclear Cu spin *I*_2_, mediated by the Cu electronic spin (*S*_2_) and effectively turned on and off by proper microwave pulses. This approach is much faster than sequential excitation of nuclear and electronic spins by combination of radio-frequency and microwave pulses.

The proposed architecture is sketched in Fig. 1. The {Cr_7_Ni} *S*_1_ = 1/2 processor is represented as a green octagon, while the nuclear copper spin *I*_2_ = 3/2 memory is depicted as a red square. This is coupled by hyperfine interaction *A* to the electronic Cu^II^ ancilla (blue circle, *S*_2_ = 1/2), which in turn is linked to the Cr_7_Ni through an exchange interaction *J*. Each elementary unit, consisting of processor, memory and ancilla, is enclosed in Fig. 1 within a blue-shaded box. Different units can be linked in a scalable platform by exploiting the power of coordination chemistry^33^ (which allows one to obtain a switchable inter-qubit coupling by means of an interposed magnetic ions^10–12^) or using coplanar resonator embedding highly concentrated magnetic fields.^13^

Herein we describe the synthesis of {Cr_7_Ni}–Cu^II^ assemblies suitable for our scheme, their characterization by electron paramagnetic resonance (EPR) and their use as quantum processors embedding error-corrected memories. We demonstrate the effectiveness of our scheme by numerical simulations of the time evolution of the system under the sequence of pulses implementing: (i) quantum gates on the processing Cr_7_Ni qubits; (ii) swap of the quantum information between processor and memory; (iii) quantum-error correction (QEC) on the nuclear Cu spin. We show that all these three steps are efficiently performed and thus make the system robust against the most harmful error in magnetic molecules, *i.e.* pure dephasing. We base our simulations on parameters measured on the molecules described, and thus show the scheme is achievable.

## Results

### Chemical engineering

The {Cr_7_Ni} rings can be included as part of [2]rotaxanes, with organic threads passing through the {Cr_7_Ni} rings. By choice of thread we can include binding groups to attach the Cu^II^ centre, and also control the interaction between {Cr_7_Ni} and Cu^II^.

**Fig. 2:**
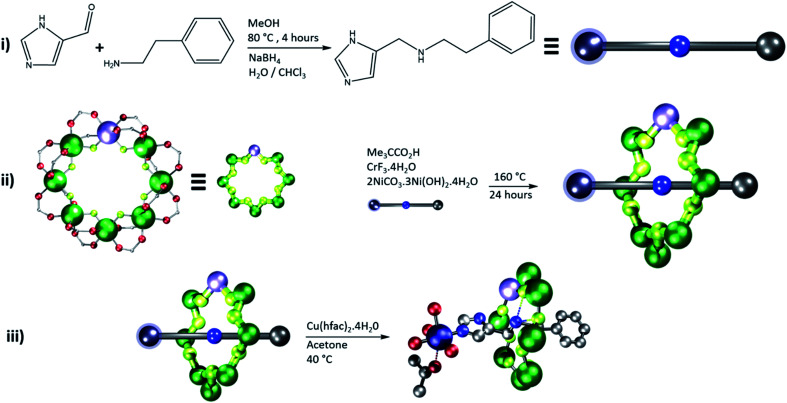
Schematic diagram to show synthesis of compounds **2**, **4**, **6**, **8** and **10**. Showing **2** as a specific example. (i) Reductive amination to form the precursor thread. (ii) Synthesis of the ring around the thread to form a [2]rotaxane, (iii) binding the Cu^II^ ion and crystallization of the product. Atom colors: blue (N), red (O), grey (C), green (Cr), lilac (Ni), navy (Cu), yellow (F).

Here we have prepared five distinct [2]rotaxanes, by initially preparing an organic thread by a Schiff-base condensation, followed by reduction (Table 1, step I in Fig. 2).^34^

**Table 1:** Threads used in [2]rotaxanes

Code	Thread
A	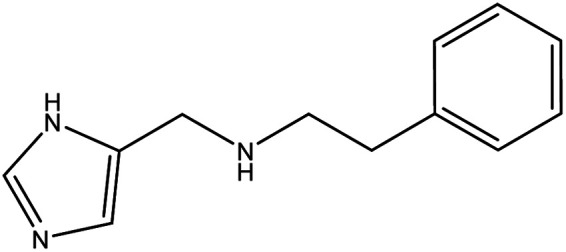
B	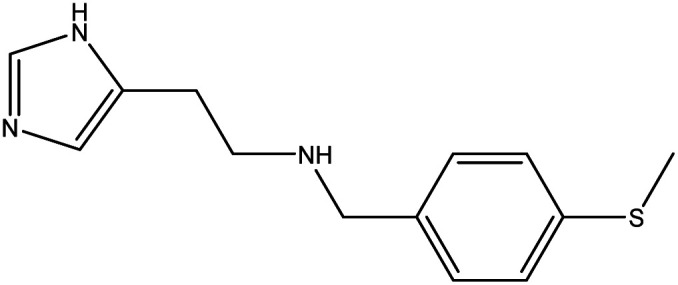
C	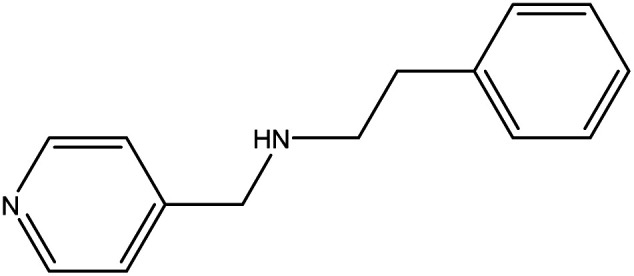
D	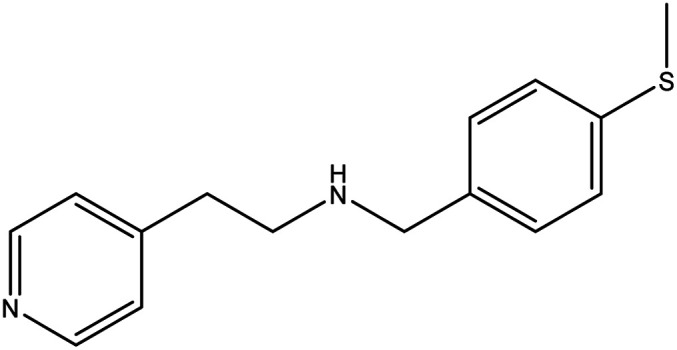
E	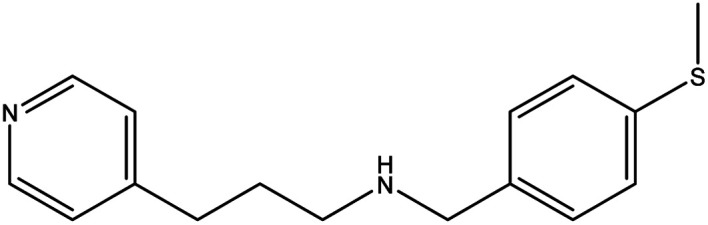

The thread is important here for two reasons: firstly it contains a secondary amine, around which the {Cr_7_Ni} ring can be grown (step II in Fig. 2), and secondly it can be used to control the distance and the pathways (bonds) that manifests the binding and exchange interaction between the {Cr_7_Ni} (*S*_1_) and Cu (*S*_2_) electron spins.

The [2]rotaxanes with threads **C** (ref. 34 and 35) and **D** (ref. 34) have been published. In all cases discussed below the {Cr_7_Ni} ring contains a regular octagon of metal centres with the divalent Ni^II^ disordered equally around the octagon. Each M…M edge is bridged by a fluoride and two carboxylate ligands. Threads **A** and **B** have imidazole head groups, whereas **C**, **D** and **E** have pyridyl head groups.

**Fig. 3:**
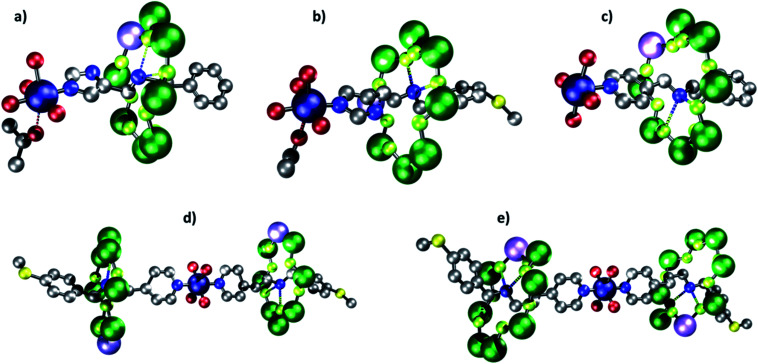
Molecular structures of rotaxanes. (a) **2**. (b) **4**. (c) **6**. (d) **8**. (e) **10**. Atom colors: blue (N), red (O), grey (C), green (Cr), lilac (Ni), navy (Cu), green-yellow (F) and yellow (S). Carboxylate groups and hydrogens omitted for clarity, dashed bonds indicate hydrogen bonds/short contacts.

All threads form [2]rotaxanes of formula {[H**X**][Cr_7_NiF_8_(O_2_C^*t*^Bu)_16_]} (**X** = **A**, **1**; **B**, **3**; **C**, **5**; **D**, **7**; **E**, **9**) (Fig. S1†). A 1 : 1 reaction of either **1** or **3** with [Cu(hfac)_2_] gives {[Cu(hfac)_2_(Me_2_CO)][**1**]} **2** and {[Cu(hfac)_2_(Me_2_CO)][**3**]} **4**; crystals can be grown from acetone. In both **2** and **4** the imidazole head-group of the thread binds *via* the nitrogen in the 3-position to the [Cu(hfac)_2_] (Fig. 3a and b). The Cu^II^ ion is six-coordinate, with a distinct elongated Jahn–Teller axis on which one hfac O-atom and an acetone molecule are bound: in **2**, the Cu–O bond lengths are 2.26(1) and 2.55(1) Å respectively; in **4**, 2.26(1) and 2.48(1) Å. The N-from the thread and three other O-atoms from hfac ligands bind in the other four sites of an octahedron, with bond lengths between 1.93(1) and 1.98(1) Å. The distances from the ammonium cation (N^am^) at the centre of the ring are given in Table 2.

**Table 2:** Cu…N^am^ distances and exchange interactions measured by EPR

Compound	Thread	Cu…N^am^ distances/Å		2*J* (cm^−1^ MHz^−1^)	Ref.
		Through bond	Through space		
**2**	A	7.71(1)	6.30(1)	−0.060/1800	This paper
**4**	B	9.13(1)	7.85(1)	−0.056/1680	This paper
**6**	C	8.99(2)	7.16(3)	−0.030/900	34 and 35
**8**	D	10.60(8)	8.59(8)	±0.010/300	31
**10**	E	11.95(9)	9.27(1)	<|0.002|/<|60|	This paper

The structures contrast with {[Cu(hfac)_2_][**5**]} **6**, which forms from the reaction of the [2]rotaxane **5** with [Cu(hfac)_2_] (Fig. 3c).^34^ In **6** the copper site is square pyramidal and the N-donor from a pyridyl-group is found in the apical position of the square pyramid. For longer threads terminated by a pyridyl-group (**D** and **E**) the composition changes and [3]rotaxanes form {[Cu(hfac)_2_][**7**])_2_} **8** and {[Cu(hfac)_2_][**9**])_2_} **10** (Fig. 3d and e). In these two molecules the [2]rotaxanes bind *vi*a the pyridyl group *trans* with respect to one another at a six coordinate Cu^II^ site. In both **8** and **10** the four shorter bonds (Cu–O and Cu–N *ca*. 1.96 and 1.98 Å respectively) are to the two pyridyl N-donors and to two O-donors from hfac *trans* to each other. Two longer Cu–O bonds (Cu…O 2.11(1) Å) are found to the other two *trans* O-donors from hfac.

### Characterization

**Fig. 4:**
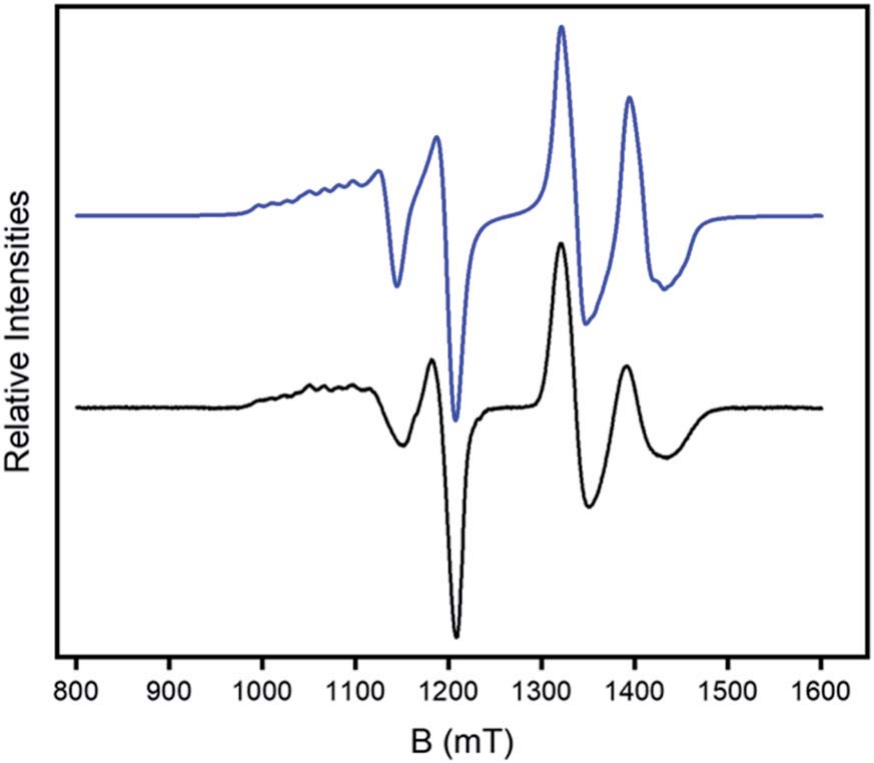
CW Q-band (*ca.* 34 GHz) EPR spectrum of **2** in a 1 : 1 CH_2_Cl_2_: toluene solution at 5 K (black) and simulation (blue).

Continuous wave (CW) Q-band EPR (*ca.* 34 GHz) spectra were measured on **2** and **4** as powders and as 3 mM solutions in dry (1 : 1) CH_2_Cl_2_ : toluene at 5 K. The spectra of **2** (Fig. 4) contains two sets of resonances. The high field features show a clear doublet at around *g* = 1.78 which is typical of the *S* = ½ signal from the {Cr_7_Ni} ring but split by an interaction with the *S* = ½ Cu^II^ ion. The low field feature is a typical axial Cu^II^ signal (*g*_*z*_ > *g*_*x*_ = *g*_*y*_) but with both features split by the interaction with the {Cr_7_Ni} ring, *i.e.* the resonance at lowest field is split into eight well resolved peaks rather than the four line pattern normally observed for a *g*_*z*_ signal from copper.

The spectra for **2** can be accurately simulated^36^ using a spin Hamiltonian of the form:

1 *H* = −2*J**S***_1_·***S***_2_ + ***S***_2_·***A**·**I***_2_ + *μ*_B_(***S***_1_·***g***_1_ + ***S***_2_·***g***_2_)·***B***

where we have considered isotropic exchange interaction (2*J*) between ***S***_1_ ({Cr_7_Ni}) and ***S***_2_ (Cu^II^), hyperfine coupling ***A*** between ***S***_2_ and ***I***_2_, and Zeeman interactions parametrized by tensors ***g***_1_ and ***g***_2_. We fix ***g***_1_ = (1.790, 1.782, 1.730), ***g***_2_ = (2.072, 2.055, 2.315) and ***A*** = (200, 200, 500) MHz from isolated {Cr_7_Ni} ring^4^ and Cu^II^ ion and the only variable parameter is the exchange coupling, *J*. Here *x*, *y*, *z* refer to the local g-frames of the two components. For ***g***_1_ ({Cr_7_Ni}) we know the *g*_*z*_ component lies perpendicular to the plane of the {Cr_7_Ni} ring.^4^ For ***g***_2_ (Cu^II^) the *z*-axis is defined by the elongated bond(s) to the Cu^II^ centre. Hence, we expect the ***g***_2_*z* component to be perpendicular to the ***g***_1_*z* component. However, as we are in the weak exchange limit and the exchange is isotropic the calculated spectrum is insensitive to the angle between the *z*-components.

The hyperfine splitting due to *A*_*z*_ is clearly visible at low field; *A*_*x*,*y*_ only produces broadening of the line around 1.2 T, which is best reproduced using *A*_*x*,*y*_ = 200 MHz. For **2** only a single exchange interaction *J* is required to simulate the spectrum well (Fig. 4).

The spectra of **4** (Fig. S2†), contains a triplet like feature at *g* = 1.78 {Cr_7_Ni} ring and the lowest field feature shows a four line hyperfine pattern at *g* = 2.315, while the resonance around *g* = 2.06 contains a central peak and two smaller peaks at either side. Two distinct coupling schemes based on Hamiltonian [1] are needed to reproduce these spectra with different *J* values. A simulation using a 2*J* = 0 (*J*_1_) reproduces the more intense peaks while a second scheme has 2*J* = −0.056 cm^−1^ (1.68 GHz) (*J*_2_), and this reproduces the less intense peaks. Three separate batches of crystals show this behaviour so it appears intrinsic to the material. The full simulation shown in Fig. S2† has the two schemes in a ratio of 4 : 1.

Our explanation of this curious result is that it is due to the dissociation of the weakly coordinated acetone ligand that is found in the crystal structure. Dissociation of the acetone would change coordination geometry and hence the Cu-based orbital that contains an unpaired electron. This could significantly change the exchange-interaction. We stress this is a working hypothesis; whatever the explanation, **4** is unsuitable for QEC.

Comparing these exchange parameters with **6**, **8** and **10** (Table 2) show that the exchange interaction between the Cu and ring can be controlled from 1800 to <60 MHz (this limit is set by considering the line-width). The magnitude of the exchange interaction |*J*| varies inversely with the distance between the two components, with the exception of **4** and **6** where the distance is very similar but the exchange interaction is twice as large in **4**. This is related to where the imidazole nitrogen binds relative to the position of the Jahn–Teller axis at Cu^II^; in **4** the imidazole nitrogen is coordinated to the copper in the *xy*-plane, where the unpaired electron is found (3d_*x*^2^−*y*^2^_^1^ configuration). In **6** the pyridine nitrogen is coordinated to the copper on the unique *z*-axis, hence approximately orthogonal to the *xy*-plane leading to a much weaker exchange.

Therefore, by choice of the thread in these rotaxanes we can vary the exchange interaction by a factor of at least thirty. We have also measured *T*_1_ and *T*_M_ times for representative examples as described previously (see ESI for details†).^34,35^ For **2** at Q-band: at 1190 mT (*i.e. g*_*xy*_ of Cu^II^) *T*_1_ = 230 and *T*_M_ = 2.0 μs; at 1337 mT (Cr_7_Ni resonance); *T*_1_ = 79 and *T*_M_ = 0.6 μs. These are very similar to previous values.

### Quantum gates and transfer of quantum information

During the implementation of complex quantum algorithms, processing qubits can remain idle (*i.e.* no gates are implemented) for rather long times between two sets of quantum operations. This is very demanding in terms of processor phase memory time (*T*_M_^*p*^). Instead, we propose to use the nuclear spin as a quantum memory in which information can be stored during these idle phases, with the help of the electronic Cu spin ancilla to transfer such information. This largely reduces the requirements on the qubit coherence, which is supplied by the much longer nuclear phase memory time *T*_M_^*n*^. As a second step, we combine this with quantum-error correction to further enhance the nuclear coherence and hence improve the performance of the quantum memory (see below).

Compounds **2** and **6** are both suitable to implement our scheme. They differ in the Cr_7_Ni–Cu exchange interaction, and we discuss the performance of our implementation as a function of *J* below. We now focus on **2**, as a representative example of this class of complexes.

**Fig. 5:**
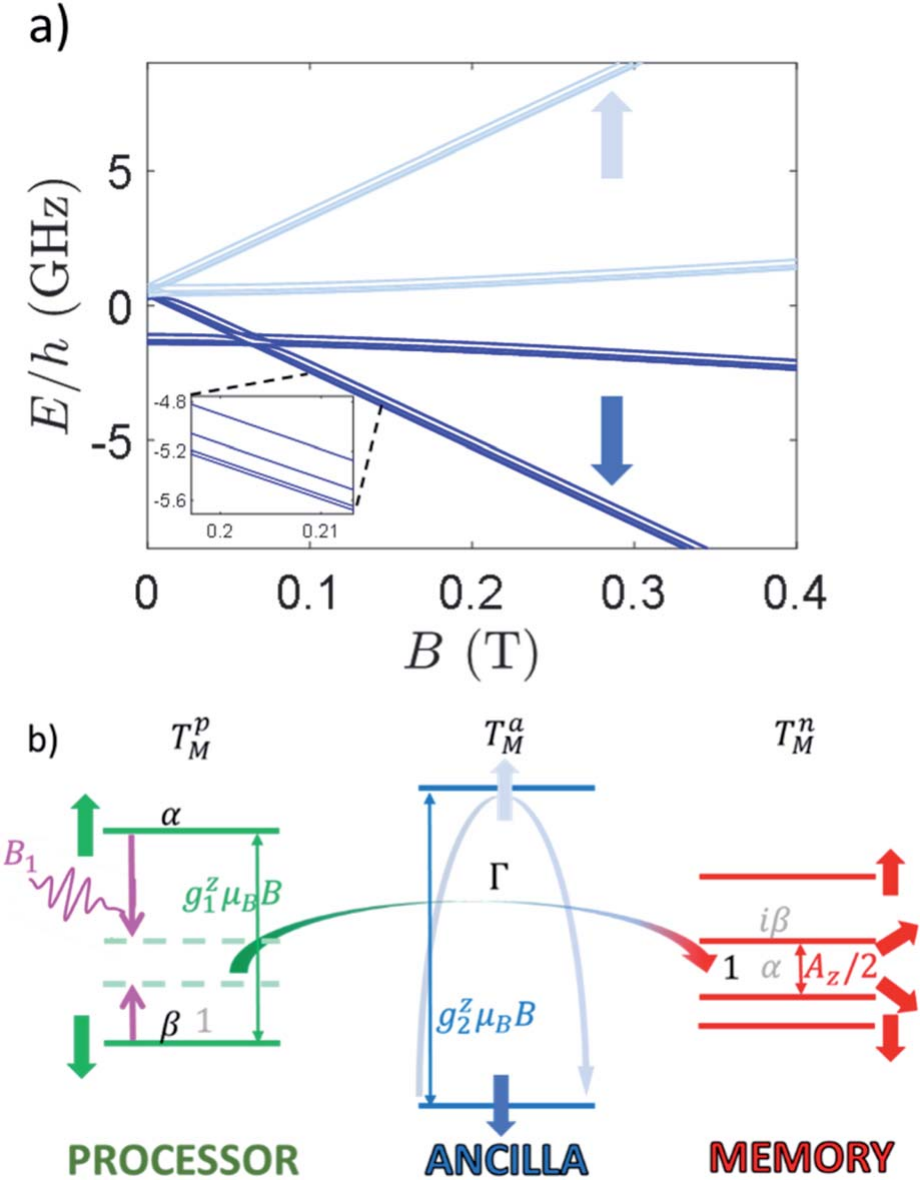
(a) Zeeman energy level diagram of **2** with a static field applied along *z* axis. Dark (light) blue levels for *B* ≳0.3 T refer to states with the ancilla froze into its |⇓(⇑)〉 state. Zoom: inset on the low-energy part, highlighting the splitting of nuclear levels induced by hyperfine and quadrupole couplings. (b) Illustrative level diagram of the three units (processor, ancilla and memory), with corresponding energy gaps to first order, in presence of a static field *B* parallel to *z*. An oscillating longitudinal field *B*_1_ (purple pulse) is used to compensate the energy mismatch (light green dashed lines) between the processor (∼*g*_1_^*z*^μ_B_*B*) and the memory (∼*A*_z_/2), thus inducing an iSWAP gate at a rate *Γ*, mediated by a virtual excitation of the ancilla (light blue arrow). The iSWAP transfers an initial superposition of the states of the processor α|−1/2〉_1_ + β|1/2〉_1_ (with memory in |−1/2〉_2_, black coefficients) into a superposition of states of the memory α|−1/2〉_2_ + *i*β|1/2〉_2_ (with processor in |−1/2〉_1_, grey coefficients).

The Zeeman energy level diagram is shown in Fig. 5a, as a function of the magnetic field *B* applied along *z*. For *B* ≳0.3–0.4 T, the state of processor, ancilla and memory are factorized, because the processor–ancilla interaction *J* and the ancilla–memory hyperfine coupling *A* are much smaller than the difference between the respective single-object excitation energies (Fig. 5b). Hence, in this regime *J*_*x*,*y*_, *A*_*x*,*y*_ do not induce any excitation on the states of the ancilla and of the memory, which are therefore “frozen”. It is then possible to manipulate the Cr_7_Ni qubit by transverse electro-magnetic pulses resonant with its gap (green levels in Fig. 5b), thus implementing quantum gates with large fidelity (see below).

The use of quantum memories requires the capability to swap quantum information back and forth from the processor to the memory on a time-scale much shorter than *T*_M_^*p*^. However, in general this could be difficult because the involved nuclear transitions are typically very slow.

Nevertheless, here we show that this swap of information can be rapidly achieved by exploiting an effective interaction (*Γ*) between processor and memory. This coupling arises from a slight mixing of the processor (Cr_7_Ni) and nuclear (Cu) wave-functions, induced by the transverse terms of the exchange (*J*_*x*,*y*_) and hyperfine (*A*_*x*,*y*_) couplings (see ESI†).

The effect of such interaction is to couple states |*m*_*s*_1__ = 1/2, *m*_*I*_2__ = −1/2〉 and |*m*_*s*_1__ = −1/2, *m*_*I*_2__ = 1/2〉. As stated above, these states are characterized by a large energy gap 
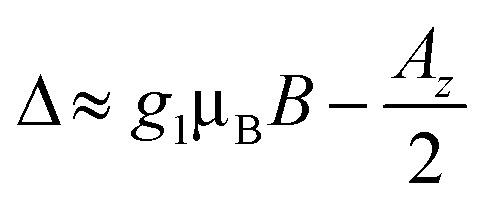
of the order of tens of GHz for the Cr_7_Ni spin *S*_1_ (in a magnetic field between 0.3 and 1.5 T), while *Γ* is ∼ tens of MHz and hence much smaller than Δ. In this regime, the coupling is effectively switched off because of the large energy mismatch. However, it can be activated when needed by using an oscillating field of frequency Δ/*ℏ* parallel to *z* to compensate the gap.^37^ Since the nuclear magnetic moment is ∼2000 times smaller than the electronic one, this uniform oscillating field practically affects only *S*_1_ and can thus be used to compensate the gap Δ and to switch on and off the iSWAP.

By choosing the proper duration of the pulse, the following transformation is implemented:|*m*_*S*_1__ = 1/2, *m*_*I*_2__ = −1/2〉 → *i*|*m*_*S*_1__ = −1/2,*m*_*I*_2__ = 1/2〉|*m*_*S*_1__ = −1/2, *m*_*I*_2__ = 1/2〉 → *i*|*m*_*S*_1__ = 1/2,*m*_*I*_2__ = −1/2〉which corresponds to the so-called iSWAP gate in quantum computing. For the specific initial state considered below, this corresponds (apart from a phase) to a flip-flop between *S*_1_ and *I*_2_ in the computational subspace (see also ESI†).

The proposed procedure has two remarkable advantages: (i) it can be switched on and off by microwave pulses. (ii) Being based on the Hamiltonian evolution, it allows us to implement the iSWAP much faster than by a combination of electronic and nuclear rotations *via* resonant transverse pulses.

We simulate all the quantum computing steps (*i.e.* the aforementioned iSWAP and processing gates acting on the qubit alone) by numerically integrating the Lindbald equation for the system density matrix *ρ*^24,38^2
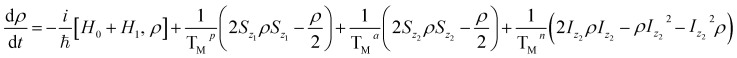
where the first term on the r.h.s. describes the coherent dynamics induced both by static (*H*) and oscillating terms (*H*_1_) in the Hamiltonian, while the others model pure dephasing on the electronic spins *S*_1,2_ = 1/2 representing processor and ancilla and on the nuclear spin *I*_2_ = 3/2.

To quantify the performance of our procedure, we compute the resulting fidelity 
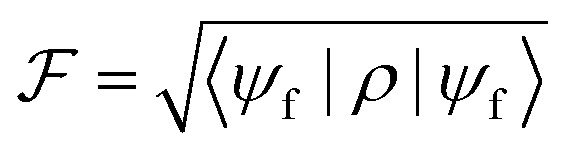
. This is a measure of the quality of the gate and represents the overlap between the final target state |*ψ*_f_〉 (corresponding to an ideal gate) and the actual system density matrix *ρ* after the pulse implementing either a qubit rotation or the iSWAP.

As an example of processing, we find ℱ = 99.9% in the simulation of the Hadamard gate. This operation maps a state with a given *m*_*S*_1__ into an equal-weight superposition, *i.e.* it implements the transformation 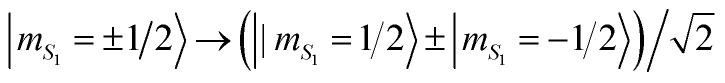. It is a very useful gates, because it is employed in many algorithms (*e.g.*, the Quantum Fourier Transform). In the simulations, we assume a phase memory time *T*_M_^*p*^ = 3 μs on the Cr_7_Ni ring, a value already measured in 2007 on a deuterated but not chemically optimized variant of Cr_7_Ni^38^ (see Fig. S8†). The effect on coherence times of exchange interactions between Cr_7_Ni rings and other magnetic ions in supra-molecular structures was found to be negligible.^11,35^ Hence, values already reported for isolated rings constitute a safe assumption for assessing the potential of the architecture.

**Fig. 6:**
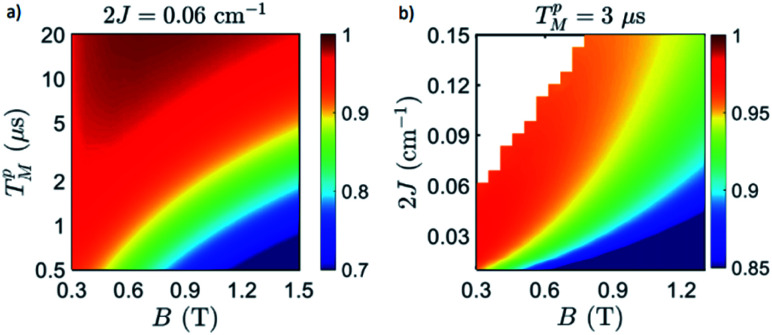
Simulated fidelity in the implementation of the iSWAP (a) as a function of external field and Cr_7_Ni phase memory time T_M_^*p*^ on complex **2** and (b) as a function of external field and of the Cu–Cr_7_Ni exchange interaction 2*J*, for fixed TM = 3 μs, as in ref. 38. Points with low factorization (<0.9) between Cr_7_Ni and Cu states have been excluded. We use an oscillating field *B*_1_ of amplitude 10 mT to reduce the duration of the gate *τ* (∝1/*B*_1_).

In compound **2** we find *T*_M_^*p*^ ∼600 ns, in line with previous measurements performed on **6**, showing *T*_M_^*p*^ ∼600–700 ns.^35^ Simulations reported below demonstrate that *T*_M_^*p*^ ∼600 ns is already enough for the implementation of the iSWAP gate (see Fig. 6 below). However, it is also important to stress that neither deuteration, neither a strategy to optimize the chemical structure to improve *T*_M_^*p*^ were applied. As already shown, reduction of the number of magnetic ions *via* deuteration can increase the phase memory time by about an order of magnitude^39^ and chemical design devoted to reduce low-energy vibrations can further increase *T*_M_^*p*^.^17^ Application of these optimization strategies will further improve the fidelity of the gate.

We now numerically investigate the implementation of the iSWAP between Cr_7_Ni processor and nuclear memory. The fidelity obtained in the simulation of the iSWAP is show in Fig. 6a as a function of the external field and of the Cr_7_Ni phase memory time *T*_M_^*p*^. As a benchmark, we use the error-prone initial state: 
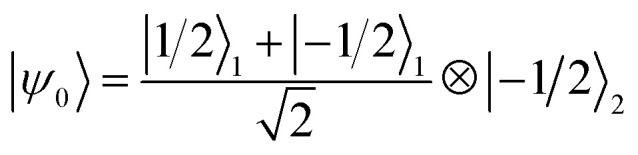
 (1 labels the Cr_7_Ni spin, 2 the Cu nuclear spin). The final target state (after the iSWAP) is: 
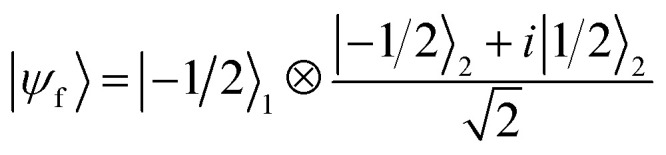
. Note that the phase *i* in the final state can be compensated by a proper *R*_*z*_ rotation, thus yielding a complete transfer of quantum information between processor and memory with no entanglement. We have checked that initializing the system in a different superposition (α|−1/2〉_1_ + β|1/2〉_1_)⊗|−1/2〉_2_ yields an analogous performance.

The behavior of ℱ is essentially determined by two competing effects. On the one hand, a large external field improves the validity of the “effective Hamiltonian” approach, thus reducing the leakage to unwanted states (*e.g.* with ancilla excited) during the implementation of the gate. On the other hand, we find that the duration of this gates (*τ*) increases quadratically with the external field *B* and is inversely proportional to the amplitude of the oscillating field *B*_1_: 
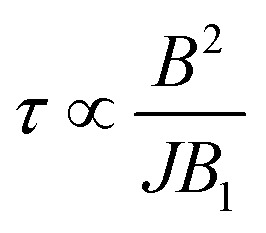
 (see ESI†). For *T*_M_^*p*^ of some μs, the detrimental effect of decoherence is more important and the optimal working point is found at rather small fields (see below). This optimal field increases with *T*_M_^*p*^. Remarkably, decoherence of both the nuclear and the electronic Cu spins does not affect the operation. Indeed, on the one hand nuclear dephasing times are much longer than the time scale of the iSWAP (*τ* ≈ hundreds of ns, see Fig. S9†). On the other hand, the electronic Cu spin only induces an effective interaction between processor and memory, without being excited (see above). Hence, the dynamics is practically unaffected by its coherence properties.

We now investigate the role of the exchange interaction. In particular, we report in Fig. 6b a simulation of the fidelity at variable field and exchange interaction, by keeping the phase memory time fixed to *T*_M_^*p*^ = 3 μs.^39^ We first note a wide region of remarkable fidelity (>0.95), which becomes very high (above 0.99) for rather small exchange interactions (0.06 cm^−1^ corresponds to complex **2**) and modest magnetic fields. By increasing *J* or decreasing *B* too much we increase the leakage of the operations and the iSWAP gate is not perfectly implemented. This effect is proportional to *J*/*B*. Conversely, the time required to implement the gate (which sets the effect of decoherence)
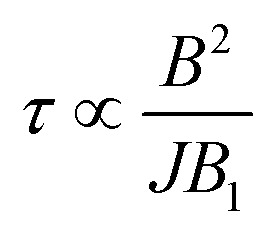
, thus making large fields unfavorable. Hence, provided that |*g*_2_^*z*^ − *g*_1_^*z*^|μ_B_*B* ≫ *J*, small values of both *J* and *B* are better.

Even larger fidelities can be obtained by improving *T*_M_^*p*^ in the 10 μs range (see ESI†), a value that was already demonstrated by chemically optimizing the molecular structure of Cr_7_Ni.^17^ We stress, however, that even a not optimized compound with *T*_M_^*p*^ ∼1 μs could be used for the first proof-of-principle experimental demonstrations, allowing one to reach ℱ >0.9 (see Fig. S10†).

### Quantum error correction

Once the information has been swapped to the memory, we apply QEC to further enhance its phase memory time. Indeed, the four nuclear spin levels of Cu^II^ can be exploited to define a memory with embedded quantum error correction (QEC) as outlined in ref. 26. Here we apply those general ideas to complex **2**, showing that it can be efficiently used to protect our quantum memory from decoherence.

To achieve this protection, we focus on pure dephasing, which represents the most important error in molecular spin systems. It was shown, by assuming a Lindbald dynamics and focusing on small *t*/*T*_2_, that pure dephasing transforms *ρ* into a mixture of the “correct state” *ρ* and of the “error state” *I*_*z*_*ρI*_*z*_, where the initial density matrix was transformed by the error operator *I*_*z*_. To protect from this form of error, one needs to identify specific superpositions of the nuclear levels which are transformed to orthogonal states by the action of this error. This can be done by choosing:3a
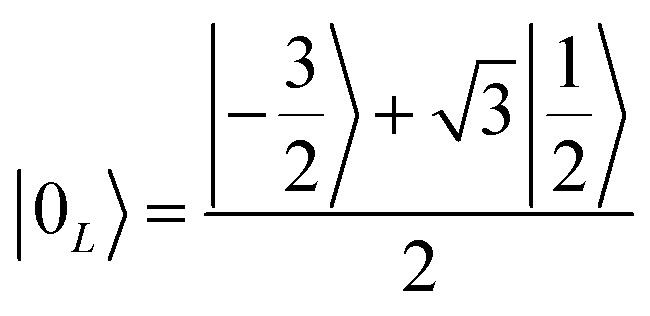
3b
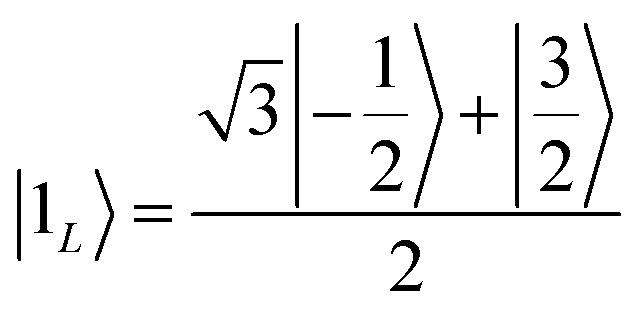


Indeed, application of the error operator *I*_*z*_ yields (apart from a normalization)
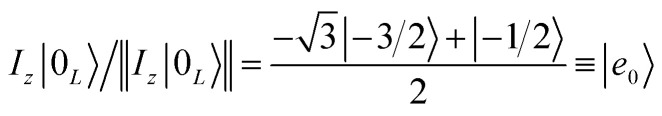

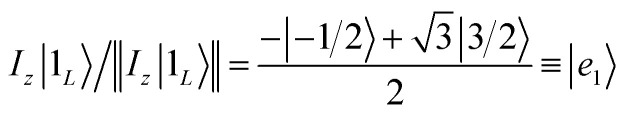


which are orthogonal to |0_*L*_〉 and |1_*L*_〉, *i.e.* 〈*e*_0_|0_*L*_〉 = 〈*e*_1_|1_*L*_〉 = 〈*e*_0_|1_*L*_〉 = 〈*e*_1_|0_*L*_〉 = 0.

We call |0_*L*_〉 and |1_*L*_〉 the “code words”, since they define our logical protected basis, and |*e*_0_〉, |*e*_1_〉 the “error words”, since they are obtained from the code words by the action of the error we aim to correct. The code-words [eqn (3a) and (3b)] also have another property: if we start from a generic superposition α|0_*L*_〉 + β|1_*L*_〉, this is brought by the error to a superposition of error-words with the same coefficients, α|*e*_0_〉 + β|*e*_1_〉. These two conditions of (i) bringing the qubit wave-function outside the computational basis and (ii) preserving the coefficients α, β of a generic superposition guarantee^32,40^ that a strategy exists to detect (i) and correct (ii) errors.

**Fig. 7:**
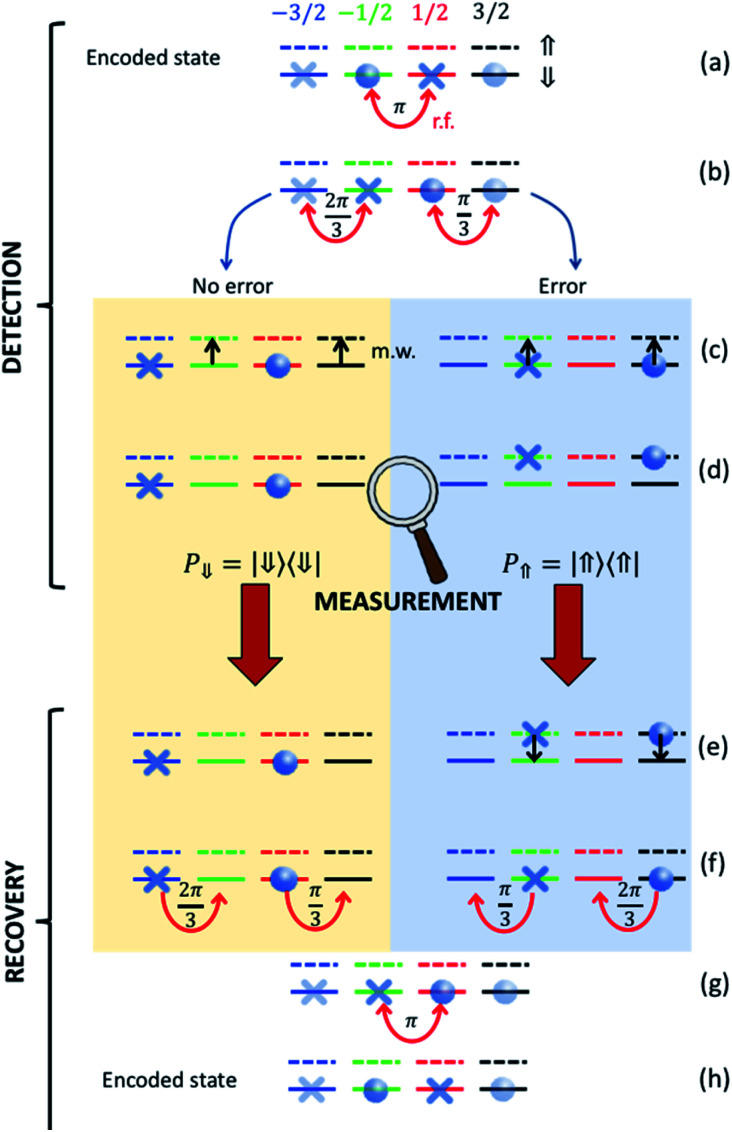
Correction procedure on nuclear spin 3/2. Starting from a generic superposition of the code words (eqn (3a) and (3b)) we first (a and b) apply r.f. pulses targeting the transitions indicated by curved red arrows (with the indicated angles). This sequence brings no error (yellow box) and error (blue) cases to orthogonal states, which then follow a different evolution when subject to the same pulse sequence. In particular, the electronic ancilla is excited by two simultaneous π m.w. pulses (black arrows) only if the nuclear spin is in |*m*_*I*_2__〉 = −1/2 and |m_*I*_2__〉 = 3/2 (c). Then a measurement of the electronic qubit (insensitive to the nuclear spin) projects either into the ⇓ or into the ⇑ manyfold (d). Only in the latter case the electronic spin is de-excited (e) and, finally, a different sequence of r.f. recovery pulses is applied, depending on the measurement outcome (f and g). The encoded state is finally recovered (h).

Our strategy is based on a sequence of radio-frequency (r.f.) and microwave (m.w.) pulses sketched in Fig. 7 and exploits excitation and measurement of the ancillary Cu^II^ electronic spin 1/2 (which is otherwise frozen into its |⇓〉 state) to discriminate the “correct” and “error state” of the nuclear qudit. For each step of the procedure, different colors in Fig. 7 indicate different nuclear states and r.f. pulses induce horizontal transitions in the ancilla |⇓〉 subspace. The corresponding levels with the ancilla excited are represented by dashed lines and vertical transitions are obtained by m.w. pulses.

After iSWAP, information has been transferred from the processor to the two |*m*_*I*_2__ = ±1/2〉 levels of the nuclear qubits, *i.e.* the system state is given by α|−1/2〉 + β|−1/2〉. A sequence r.f. pulses^26^ is then used to transform this state to the encoded superposition α|0_*L*_〉 + β|1_*L*_〉. We then wait for a memory time *t* before implementing the correction procedure. This begins by applying r.f. pulses to bring each code/error word to a well-defined |*m*_*I*_2__〉 state (Fig. 7a and b), *i.e.* we implement the transformation.α|0_*L*_〉 + β|1_*L*_〉 → α|−3/2〉+β|1/2〉α|*e*_0_〉 + β|*e*_1_〉 →α|−1/2〉+β|3/2〉

Then, we conditionally excite the Cu^II^ electronic spin 1/2 only if the nuclear qudit is in |*m*_*I*_2__ = −1/2〉 and |*m*_*I*_2__ = 3/2〉. This is achieved by two simultaneous m.w. π pulses resonant with transitions |*m*_*I*_2__ = −1/2⇓〉→|*m*_*I*_2__ = −1/2⇑〉 and |*m*_*I*_2__ = 3/2⇓〉→|*m*_*I*_2__ = 3/2⇑〉 (Fig. 7c). These excitations are made distinguishable by the hyperfine interaction *A*_*z*_. In the next step, we measure the state of the electronic ancilla, without affecting the nuclear spin (d). Only in case of positive outcome, we de-excite the ancilla (e); finally, we apply different r.f. pulses depending on the measurement result (Fig. 7f and g) to recover the correct encoded state (h).

**Fig. 8:**
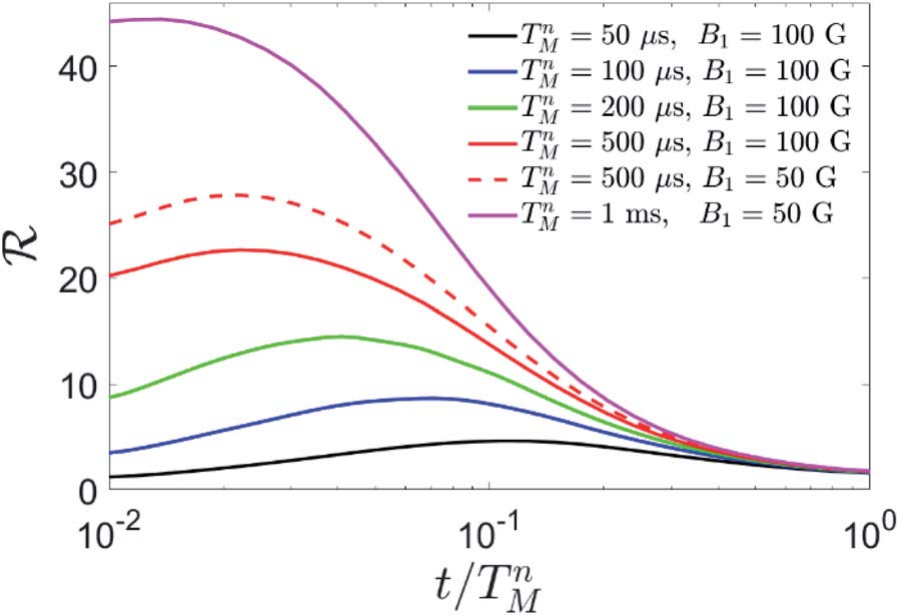
Simulation of QEC on Cu quantum memories. Gain ratio *ℛ* = *ℇU*/*ℇ* after QEC with respect to an uncorrected processing qubit, for different values of the nuclear phase memory time *T*_M_^*n*^ and of the pulse amplitude *B*_1_. In the simulation, performed on compound **2** at *B* = 0.3 T, we have used *T*_M_^*p*^ = 3 μs and we have included a nuclear quadrupole *p* = 50 MHz, reasonable for the dominant ^63^Cu isotope,^41^ and the measured phase memory time of *T*_M_^*a*^ = 2 μs on the electronic Cu spin.

We apply this scheme to complex **2**, by numerically solving the Lindblad equation for the system density matrix. We include pure dephasing in all the steps of the simulation, namely encoding, memory time and correction. We quantify the performance of our approach by computing the error *ε* = 1 − ℱ^2^ after QEC onto the nuclear spin. This is compared to the error *ε*_*U*_ accumulated during the same amount of time by an uncorrected processing qubit, subject to pure dephasing with phase memory time *T*_M_^*p*^. As a figure of merit, we compute the gain ratio *ℛ* = *ε*_*U*_/*ε* as a function of the memory time *t*, in units of the nuclear phase memory time *T*_M_^*n*^. Results are shown in Fig. 8. Since we do not have an experimental determination of *T*_M_^*n*^, we compare the performance of the QEC algorithm for different *T*_M_^*n*^ and experimental conditions.

We can, nevertheless, fix a range of investigation by considering the measured value of the electronic coherence *T*_M_^*a*^ and the ratio of nuclear to electronic coherence times measured in other molecular complexes. In particular, nuclear-magnetic resonance experiments on Yb(trensal)^25^ and VOTPP^42^ show *T*_M_^*n*^ values up to 50 times larger than their electronic counterpart, at magnetic fields of about 0.3 T. Here we find *T*_M_^*a*^ = 2 μs for the electronic phase memory time, where, however, the sample was neither deuterated nor chemically optimized to reduce decoherence. Hence, one could expect a nuclear phase memory time of at least ∼100 μs, but potentially much more. We therefore examine the result of our QEC procedure for 50 μs ≤ *T*_M_^*n*^ ≤ 1 ms. We find an important reduction of the error in all the range.

In particular, for small values of *T*_M_^*n*^ <200 μs the gain is essentially due to the storage of information in the nuclear spins, while QEC is ineffective. This is due to pure dephasing and gate errors (induced by finite duration and band-width of the pulses) acting also during the QEC procedure, which requires a certain amount of time to be implemented. Errors accumulated during this step cannot be corrected. However, the larger *T*_M_^*n*^, the smaller is the effect of the finite duration of the correction procedure on *ε*. Hence, for *T*_M_^*n*^ >200 μs QEC becomes advantageous, *i.e.* the error after QEC is smaller than that accumulated by a nuclear spin ½ during the same memory time. In this rather small *T*_M_^*n*^ regime, gate errors are negligible, compared to dephasing errors. Hence, we choose to apply r.f. pulses of amplitude 10 mT in order to reduce as much as possible the duration of the gates, thus limiting the effect of dephasing. For *T*_M_^*n*^ = 500 μs, we find a remarkable gain *ℛ* >20. This gain is a measure of the effectiveness of our error mitigation strategy. Now, dephasing errors are significantly reduced by the large value of *T*_M_^*n*^, thus making gate errors more relevant. Hence, we find it advantageous to use longer pulses, *i.e.* to reduce the amplitude of the oscillating field from 10 to 5 mT (dashed *vs.* continuous red line in Fig. 8). This makes pulses more selective in frequency, thus limiting leakage to other states when a single pair of levels is resonantly addressed. Finally, for *T*_M_^*n*^ = 1 ms, the gain overcomes 40.

It is also important to note that large values of ℛ are generally obtained for memory times ∼0.1 *T*_M_^*n*^. These values of *t* represent the optimal repetition times to apply QEC cycles, thus constituting an important figure of merit of the performance of the procedure, besides *ℛ*.

## Discussion and conclusions

We have presented a family of supra-molecular Cr_7_Ni–Cu complexes which could constitute the elementary units of a scalable architecture for quantum information processing. Indeed, each molecule embeds both a processing unit (represented by the Cr_7_Ni ring) and a quantum memory (the nuclear Cu spin 3/2) naturally supporting quantum error correction. We have demonstrated the remarkable performance of our platform in (i) processing quantum information, (ii) swapping it back and forth from the processor to the memory and (iii) fighting decoherence by a targeted QEC procedure on the memory, an effect which could be made even larger by using a larger spin memory.^26^ These tasks are achieved by exploiting the electronic Cu spin ½ as an ancilla, whose coherence properties do not affect our scheme. The proposed architecture exploits the best characteristics of the different units: on the one hand the fast manipulations of electronic spin processors and chemical flexibility of Cr_7_Ni rings, on the other hand the intrinsic protection and isolation from the environment provided by nuclear spins. Keeping memories separated from the processors further protects the stored information from errors, while reducing the requirements on the coherence times of the processors and hence making our proposal achievable in the short-term.^30,31^ Conversely, implementation of a fully fault-tolerant architecture (in which quantum operations are implemented on quantum error corrected units) would pose much more stringent requirements.^28,29^

Thanks to the impressive flexibility of coordination chemistry, the molecules presented here could be used to realize a scalable device;^33^ chains of poly-rotaxanes can be synthesized, linked through rings and/or through the thread with blocking groups. This could be the basis of the scalable architecture sketched in Fig. 1, in which different units are linked either through rings (with a rotaxane thread anchoring a memory to each Cr_7_Ni ring) or through the thread (with a Cu memory connected to the Ni within each ring), as already demonstrated in compound **10**. In both cases, the presence of an interposed magnetic ion between the rings belonging to different units^10,11^ could be used to mediate a switchable interaction between the qubits. This would provide all the necessary ingredients for a scalable molecular quantum processor.

Implementation of the proposed scheme requires to alternate pulses parallel and perpendicular to the external field. A possibility is to use oscillating fields at an intermediate angle between 0 and 90° with respect to the static field. Indeed, at each frequency (*i.e.*, the qubit one and the one used for iSWAP) only the component with the right orientation makes effect. This could be achieved with a non-standard EPR setup containing a dual-mode cavity and two microwave sources.

## Data availability

We have deposited the crystallographic data.

## Author contributions

SJL carried out most of the synthetic work described here, advised by GAT with some assistance from TSB. AC performed the theoretical analysis and calculations. EPR spectra were recorded by SJL, advised by EJLMcI. SC and REPW devised the project. The manuscript was written by AC, SJL, SC and REPW with input from all authors.

## Conflicts of interest

There are no conflicts to declare.

## Supplementary Material

SC-012-D1SC01506K-s001

SC-012-D1SC01506K-s002
