# Supplementary material for: Targeting molecular quantum memory with embedded error correction
Source: Chem Sci. 2021 Jun 2;12(26):9104–13. doi: 10.1039/d1sc01506k (PMC8261727; doi:10.1039/d1sc01506k)
Supplement: SC-012-D1SC01506K-s001 [file SC-012-D1SC01506K-s001.pdf]

Electronic Supplementary Material (ESI) for Chemical Science.
This journal is © The Royal Society of Chemistry 2021

Electronic Supplementary Information: Targeting Molecular Quantum Memory with Embedded Error Correction

Selena J. Lockyer,[a] Alessandro Chiesa,[b,c] Grigore A. Timco,[a] Eric J. L. McInnes,[a] Thomas Bennett,[a] Inigo Vitorica-Yrzebal,[a] Stefano Carretta[b,c] and Richard E. P. Winpenny[a]

[a]*Department of Chemistry and Photon Science Institute, The University of Manchester, Oxford Road, Manchester M13 9PL, UK.*

[b]*Università di Parma, Dipartimento di Scienze Matematiche, Fisiche e Informatiche, I-43124 Parma, Italy*

[c]*UdR Parma, INSTM, I-43124 Parma, Italy*

Contents:

Experimental Section **S3**

Organic Thread Synthesis **S3**

Heterometallic Rotaxane Synthesis **S4**

Crystallography Details **S7**

Table S1: Crystallographic Information for **2**, **4** and **10** **S8**

Structures **S9**

Figure 1: Structures of the [2]rotaxanes **1**, **3**, **5**, **7** and **9** **S9**

EPR Details and Measurements **S10**

Figure 2: CW Q-Band Powder EPR for **2** and **4** **S10**

Figure 3: CW Q-Band J coupling simulations **4** **S11**

Figure 4: CW Q-Band EPR spectra for **10** **S12**

Figure 5: Echo detected field sweep for **2** **S13**

Figure 6: Phase memory times (T_m) for **2** **S14**

Figure 7: Spin lattice relaxation times (T_1) for **2** **S15**

Computational Quantum Simulation and Processing Figures **S16**

Figure S8: Simulation of the Hadamard gate on the processing unit **S17**

Figure S9: Duration of the iSWAP **S17**

Figure S10: Simulation of iSWAP **S18**

SI References **S19**

Experimental section:

General remarks: All starting reagents and materials were sourced from Sigma-Aldrich, Alfa and/or Fluorochem. Unless stated otherwise, all reagents and solvents were used without further purification. The syntheses of the hybrid organic-inorganic rotaxanes were carried out in Erlenmeyer Teflon® FEP flasks supplied by Fisher. Column chromatography was performed using either 40-63 µm silica from Sigma-Aldrich or a Grace Reverelis ® X2 Autocolumn with Grace Reverelis ® NP cartridges. Chemical shifts are reported in parts per million (ppm) from low to high frequency and referenced to the residual solvent resonance. ESI mass spectrometry and microanalysis were carried out by the services at The University of Manchester.

1. Organic Thread Synthesis (R,R'NH): All threads were prepared using reductive amination - Schiff base condensation methods.[S1] **Thread C** and **Thread D** were prepared as per previously published [S2]

1.1 Thread A ($ImCH_2NHCH_2CH_2C_6H_5$): A solution of phenethylamine (0.962 mL, 10 mmol) and 4-Imidazolecarboxaldehyde (1 g, 10 mmol) in methanol (30 mL) was refluxed for 5 hr under an N_2 atmosphere, then stirred at room temperature for 3 hr. Excess $NaBH_4$ (1.52 g, 40 mmol) was added and the reaction mixture was stirred for 2 hr. The reaction was then quenched with water (20 mL) and the residue was extracted with chloroform (3 x 25 mL). The chloroform extracts were dried ($MgSO_4$) and the solvents evaporated under reduced pressure to leave a yellow oil. Yield: 1.58 g, 80%. ^1H NMR (400 MHz, 293 K, CDCl3) δ 7.58 (s, 1H), 7.38-7.25 (m, 5H), 6.92 (s, 1H), 3.86 (s, 2H), 2.99 (t, 2H), 2.89 (t, 2H); ESI MS m/z (relative intensity) 202.3 $[M+H]^+$, 224.3 $[M+Na]^+$.

1.2 Thread B ($ImCH_2CH_2NHCH_2C_6H_5SCH_3$): A solution of 4-(methylthio)benzaldehyde (0.47 mL, 4.5 mmol) and histamine (0.5 g, 4.5 mmol) in methanol (30 mL) was refluxed for 5 hr under an N_2 atmosphere, then stirred at room temperature for 3 hr. Excess $NaBH_4$ (0.68 g, 18 mmol) was added and the reaction mixture was stirred for 2 hr. The reaction was then quenched with water (20 mL) and the residue was extracted with chloroform (3 x 25 mL). The chloroform extracts were dried ($MgSO_4$) and the solvents evaporated under reduced pressure to leave a light orange / yellow oil. Yield: 0.87 g, 79%. ^{1} H NMR (400 MHz, 293 K, CDCl3) δ 7.47 (s, 1H), 7.26-7.13 (m, 4H), 6.74 (s, 1H), 2.87 (t, 2H), 2.74 (t, 2H), 2.43 (s, 3H); ESI MS m/z (relative intensity) 249.4 M+H]$^+$, 272.4 [M+Na]$^+$.

1.3 Thread E ($pyCH_2CH_2CH_2NHCH_2C_6H_4SCH_3$): A solution of 4-(methylthio)benzaldehyde (0.95 mL, 4.5 mmol) and histamine (1.0 g, 4.5 mmol) in methanol (30 mL) was refluxed for 5 hr under an N_2 atmosphere, then stirred at room temperature for 3 hr. Excess $NaBH_4$ (0.68 g, 18 mmol) was added and the reaction mixture was stirred for 2 hr. The reaction was then quenched with water (20 mL) and the residue was extracted with chloroform (3 x 25 mL). The chloroform extracts were dried ($MgSO_4$) and the solvents evaporated under reduced pressure to leave a viscous yellow oil. Yield: 0.87 g, 79%. ^{1}H NMR (400 MHz, 293 K, CDCl3) δ 8.50 (d, 2H), 7.29-7.35 (m, 2H), 7.22-7.27 (m, 2H) 7.09-7.13 (m, 2H), 3.79 (s, 2H), 2.69 (m, 4H), 2.49 (s, 3H), 1.91 (q, 2H); ESI MS m/z (relative intensity) 244.2 [M+H]$^+$, 267.2 [M+Na]$^+$.

2.0 Heterometallic Rotaxane Synthesis: ($R,R'NH_2$)[$Cr_7NiF_8(Piv)_{16}$]; **5**, **6**, **7** and **8** were prepared as per previously published [S2]

2.1 ($ImCH_2NH_2CH_2CH_2C_6H_5$)[$Cr_7NiF_8(O_2C^tBu)_{16}$] **1**: Me_3CCO_2H (25 g, 245 mmol), $CrF_3.4H_2O$ (3 g, 16 mmol), and thread **A** (0.50 g, 2.5 mmol) were added to a Teflon flask and heated at 140 ^{o}C for 30 minutes. $2NiCO_3.3Ni(OH)_2.4H_2O$ (0.35 g, 0.6 mmol) was added and the melt heated at 140 ^{o} C for a further 60 minutes. The heat was then increased to 160 ^{o}C for 22 hours. The mixture was left to cool to room temperature, 35 mL acetonitrile was added, stirred for 1 hr, then filtered. The isolated green powder was washed with acetonitrile. Column chromatography (10:1 toluene:ethyl acetate) produced two fractions, the second one

containing the product. Solvents were removed to produce a dry green powder. Yield: 2.7 g, 47%. ESI MS m/z (relative intensity) 2395$[M+H]^+$, 2417 $[M+Na]^+$. Elemental analysis %: calc. for $C_{92}H_{158}Cr_7CuF_8N_3NiO_{32}$: Cr 15.21, Ni 2.45, C 46.18, H 6.66, N 1.76; found: Cr 14.59, Ni 2.44, C 47.15, H 6.83, N 1.75.

2.2 $\{[Cu(hfac)_2OCMe_2][ImCH_2NH_2CH_2CH_2C_6H_5)[Cr_7NiF_8(O_2C^tBu)_{16}]\}$ **2**: To a warm solution (~50 ^oC) of **1** (0.25 g, 0.1 mmol) in acetone (20 mL), copper(II) hexafluoroacetylacetonate hydrate (0.05 g, 0.1 mmol) was added and the solution stirred for 10 minutes, filtered and then left to cool in a sealed flask at room temperature. Dark green crystals suitable for single crystal X-ray diffraction formed slowly over 24 hours. The crystals were separated by filtration and washed with acetone, then acetonitrile. Yield: 0.50 g (22%). Elemental analysis %: calc. for $C_{102}H_{161}Cr_7CuF_{20}N_3NiO_{36}$: Cr 12.68, Ni 2.04, Cu 2.21, C 42.66, H 5.65, N 1.46; found: Cr 12.36, Ni 2.10, Cu 2.13, C 42.76, H 5.74, N 1.47.

2.3 $(ImCH_2CH_2NH_2CH_2C_6H_5SCH_3)[Cr_7NiF_8(O_2C^tBu)_{16}]$ **3**: Me_3CCO_2H (25 g, 245 mmol), $CrF_3.4H_2O$ (3 g, 16 mmol), and thread **B** (0.62 g, 2.5 mmol) were added to a Teflon flask and heated at 140 ^oC for 30 minutes. $2NiCO_3.3Ni(OH)_2.4H_2O$ (0.35 g, 0.6 mmol) was added and the melt heated at 140 ^{o} C for a further 60 minutes. The heat was then increased to 160 ^oC for 22 hours. The mixture was left to cool to room temperature, 35 mL acetonitrile was added, stirred for 1 hr, then filtered. The isolated green powder was washed with acetonitrile. Column chromatography (10:1 toluene:ethyl acetate) produced three fractions, the third one containing the product. Solvents were removed to produce a dry green powder. Yield: 1.95 g, 33%. ESI MS m/z (relative intensity) 2439$[M+H]^+$, 2462 $[M+Na]^+$. Elemental analysis %: calc. for $C_{93}H_{161}Cr_7F_8N_3NiO_{32}S$: Cr 14.92, Ni 2.41, C 45.78, H 6.65, N 1.72, S 1.31; found: Cr 13.92, Ni 2.37, C 46.46, H 6.83, N 1.69, S 1.26.

2.4 $\{[Cu(hfac)_2OCMe_2][ImCH_2CH_2NH_2CH_2C_6H_5SCH_3)[Cr_7NiF_8(O_2C^tBu)_{16}]\}$ **4**: To a warm solution (~40 ^oC) of **3** (0.5 g, 0.20 mmol) in acetone (30 mL), copper(II) hexafluoroacetylacetonate hydrate (0.1 g, 0.20 mmol) was added and the solution stirred for 10 minutes, filtered and then left to cool in a sealed flask at room temperature. Dark green crystals suitable for single crystal X-ray diffraction formed slowly over 48 hours. The crystals were separated by filtration and washed with acetone, then acetonitrile. Yield: 0.40 g (66%). Elemental analysis %: calc. for

$C_{103}H_{163}Cr_7CuF_{20}N_3NiO_{36}S$: Cr 12.47, Ni 2.01, Cu 2.18, C 42.40, H 5.63, N 1.44, S 1.10; found: Cr 12.17, Ni 2.10, Cu 2.16, C 42.72, H 5.78, N 1.46, S 1.10.

2.5 $(pyCH_2CH_2CH_2NHCH_2C_6H_4SCH_3)[Cr_7NiF_8(O_2C^tBu)_{16}]$ **9**: Me_3CCO_2H (25 g, 245 mmol), $CrF_3.4H_2O$ (3 g, 16 mmol), and thread **C** (0.50 g, 1.8 mmol) were added to a Teflon flask and heated at 140 ^oC for 30 minutes. $2NiCO_3.3Ni(OH)_2.4H_2O$ (0.35 g, 0.6 mmol) was added and the melt heated at 140 ^{o} C for a further 60 minutes. The heat was then increased to 160 ^oC for 22 hours. The mixture was left to cool to room temperature, 35 mL acetonitrile was added, stirred for 1 hr, then filtered. The isolated green powder was washed with acetonitrile. Column chromatography (10:1 toluene:ethyl acetate) produced two fractions, the second one containing the product. Solvents were removed to produce a dry green powder. Yield: 0.6 g, 13%. ESI MS m/z (relative intensity) 2466$[M+H]^+$, 2488 $[M+Na]^+$, 2504 $[M+K]^+$. Elemental analysis %: calc. for $C_{96}H_{165}Cr_7F_8N_2NiO_{32}S$: Cr 14.76, Ni 2.38, C 46.76, H 6.74, N 1.14; found: Cr 13.09, Ni 2.15, C 47.61, H 7.07, N 1.04.

2.6 $\{[Cu(hfac)_2]([pyCH_2CH_2CH_2NHCH_2C_6H_4SCH_3)[Cr_7NiF_8(O_2C^tBu)_{16}])_2\}$ **10**: To a warm solution (~30 ^oC) of **9** (0.5 g, 0.20 mmol) in acetone (30 mL) and dichloromethane (1 mL), copper(II) hexafluoroacetylacetonate hydrate (0.1 g, 0.20 mmol) was added and the solution stirred for 10 minutes, filtered and then left to cool in a sealed flask at room temperature. Dark green crystals suitable for single crystal X-ray diffraction formed slowly over 24 hours. The crystals were separated by filtration and washed with acetone, then acetonitrile. Yield: 0.25 g (42%). Elemental analysis %: calc. for $C_{202}H_{332}Cr_{14}CuF_{28}N_4Ni_2O_{68}S_2$: Cr 13.463, Ni 2.17, Cu 1.17, C 44.85, H 6.19, N 1.04, S 1.19; found: Cr 12.83, Ni 2.30, Cu 1.18, C 44.69, H 6.26, N 1.01, S 1.19.

Crystallography details

Data Collection. X-Ray data for compound in **2**, **4** and **10** were collected at a temperature of 100 K using a Rigaku FR-X with Cu-Kα radiation equipped with a HypixHE6000 detector, equipped with an Oxford Cryosystems nitrogen flow gas system. Data was measured using CrysAlisPro suite of programs.

Crystal structure determinations and refinements. X-Ray data were processed and reduced using CrysAlisPro suite of programmes. Absorption correction was performed using empirical methods (SCALE3 ABSPACK) based upon symmetry-equivalent reflections combined with measurements at different azimuthal angles.[S3] The crystal structure was solved and refined against all F^2 values using the SHELXL and Olex 2 suite of programmes.[S4] Despite the highly intense X-ray source, crystals of **2** and **10** present a diffraction limit of 1.09 Å, and 1 Å respectively.

All atoms in crystal structures **4** were refined anisotropically with the exception of the hydrogens atoms. In the crystal structure **2** and **10**, only the non-disordered toms were refined anisotropically in order to keep the highest data/parameters ratio. Hydrogen atoms were placed in the calculated idealized positions for all crystal structures. The pivalate ligands, threads and hfac ligands in crystal structures **2** and **10** were disordered and modelled over two positions, using structural same distance (SADI) and distance fix (DFIX) Shelxl restraints commands. The atomic displacement parameters (adp) of the ligands have been restrained using similar Ueq and rigid bond (SIMU) and Similar Ueq (SIMU) restraints.

DCM molecules in compound **10** were also disordered and modelled over two positions.

Compounds **2** present large voids filled with featureless electron density. Squeeze software implemented in Platon shows an electron count of 264 electrons which correspond to one molecule of acetone in the asymmetric unit.

A number of A and B alerts were found, especially for structure **2** and **10**, due to the crystal poor resolution obtained for the two crystal structures. Unfortunately, this resolution is common in large molecules with large intermolecular spaces filled with disordered solvent molecules or large amount of disorder.

CCDC 2057528-2057530 contains the supplementary crystallographic data for this paper. These data can be obtained free of charge *via* www.ccdc.cam.ac.uk/conts/retrieving.html (or from the Cambridge Crystallographic Data Centre, 12 Union Road, Cambridge CB21EZ, UK; fax: (+44)1223-336-033; or deposit@ccdc.cam.ac.uk).

Table S1. Crystallographic information for **2**, **4** and **10**

Identification code	**2**	**4**	**10**
Empirical formula	$C_{102}H_{162.41}Cr_8CuF_{20}N_3O_{36.2}$	$C_{108.2}H_{173.6}Cr_7CuF_{20}N_3NiO_{37.73}S$	$C_{206}H_{328}Cl_{12}Cr_{12.56}CuF_{28}N_4Ni_2O_{68}S_2$
Formula weight	2869.55	3018.49	5804.30
Temperature/K	100	100.00(10)	100.0(7)
Crystal system	orthorhombic	monoclinic	monoclinic
Space group	Pbca	$P2_1/n$	$P2_1/n$
a/Å	28.1451(14)	20.38800(10)	20.4253(11)
b/Å	28.4820(15)	29.9713(2)	30.2613(12)
c/Å	36.1611(9)	24.0737(2)	23.6931(13)
α/°	90	90	90
β/°	90	90.4360(10)	90.912(5)
γ/°	90	90	90
Volume/Å^3	28988(2)	14709.92(17)	14642.8(13)
Z	8	4	2
ρ_{calc}g/cm^3	1.315	1.363	1.316
μ/mm^{-1}	5.703	5.332	5.755
F(000)	11888.0	6267.0	6021.0
Crystal size/mm^3	0.3 × 0.3 × 0.1	0.2 × 0.2 × 0.1	0.15 × 0.12 × 0.06
Radiation	CuKα (λ = 1.54184)	CuKα (λ = 1.54184)	Cu Kα (λ = 1.54184)
2Θ range for data collection/°	4.888 to 90.026	4.708 to 152.622	4.738 to 100.872
Index ranges	-23 ≤ h ≤ 25, -24 ≤ k ≤ 26, -33 ≤ l ≤ 22	-25 ≤ h ≤ 22, -33 ≤ k ≤ 37, -30 ≤ l ≤ 30	-20 ≤ h ≤ 20, -26 ≤ k ≤ 30, -23 ≤ l ≤ 21
Reflections collected	38107	89150	53536
Independent reflections	11313 [R_{int} = 0.1023, R_{sigma} = 0.0780]	29953 [R_{int} = 0.0415, R_{sigma} = 0.0459]	14501 [R_{int} = 0.1355, R_{sigma} = 0.1082]
Data/restraints/parameters	11313/719/1411	29953/1274/1939	14501/975/1763
Goodness-of-fit on F^2	1.116	1.048	1.042
Final R indexes [I>=2σ (I)]	R_1 = 0.0779, wR_2 = 0.2275	R_1 = 0.0581, wR_2 = 0.1530	R_1 = 0.0919, wR_2 = 0.2485
Final R indexes [all data]	R_1 = 0.1015, wR_2 = 0.2469	R_1 = 0.0717, wR_2 = 0.1621	R_1 = 0.1578, wR_2 = 0.2936
Largest diff. peak/hole / e Å^{-3}	1.27/-1.01	1.03/-0.87	1.47/-0.69

The crystal structures and refinement details for **6** and **8** have been previously published.[S2]

Structures:

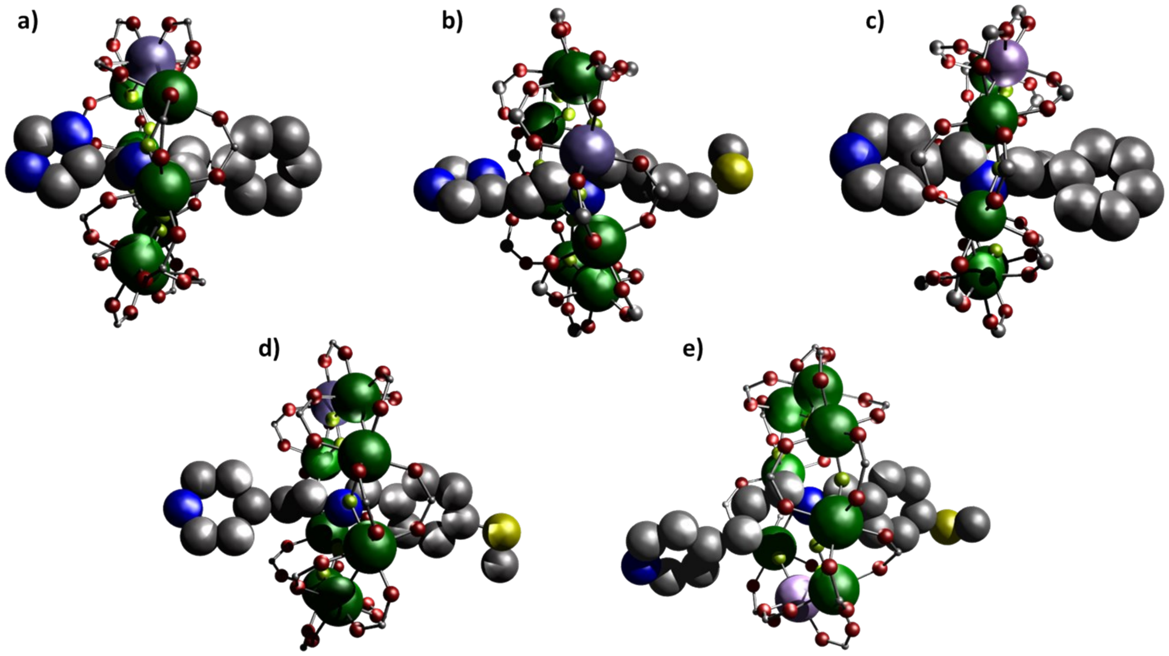

Fig. S1 Crystal structures of [2]rotaxanes. a) **1**, b) **3**, c) **5**, d) **7** and e) **9**. Atom colors: blue (N), red (O), grey (C), green (Cr), lilac (Ni), yellow (F) and dull yellow (S). ^{t}Bu(pivalate) groups and hydrogens omitted for clarity.

EPR details and measurements:

Continuous wave Q-band (~34 GHz) and X-band (~9.5 GHz) EPR spectra were recorded with a Bruker EMX580 spectrometer. The continuous wave data were collected on polycrystalline powders and a solution of 1:1 toluene / DCM at 5 K (unless otherwise stated) using liquid helium cooling. All continuous wave spectra were field corrected using a 'Strong Pitch' standard (g = 2.0028) and all powder samples were checked for any polycrystalline nature, by measuring multiple random rotations.

Spectral simulations were performed using the EasySpin 5.2.25 simulation software[S5] unless

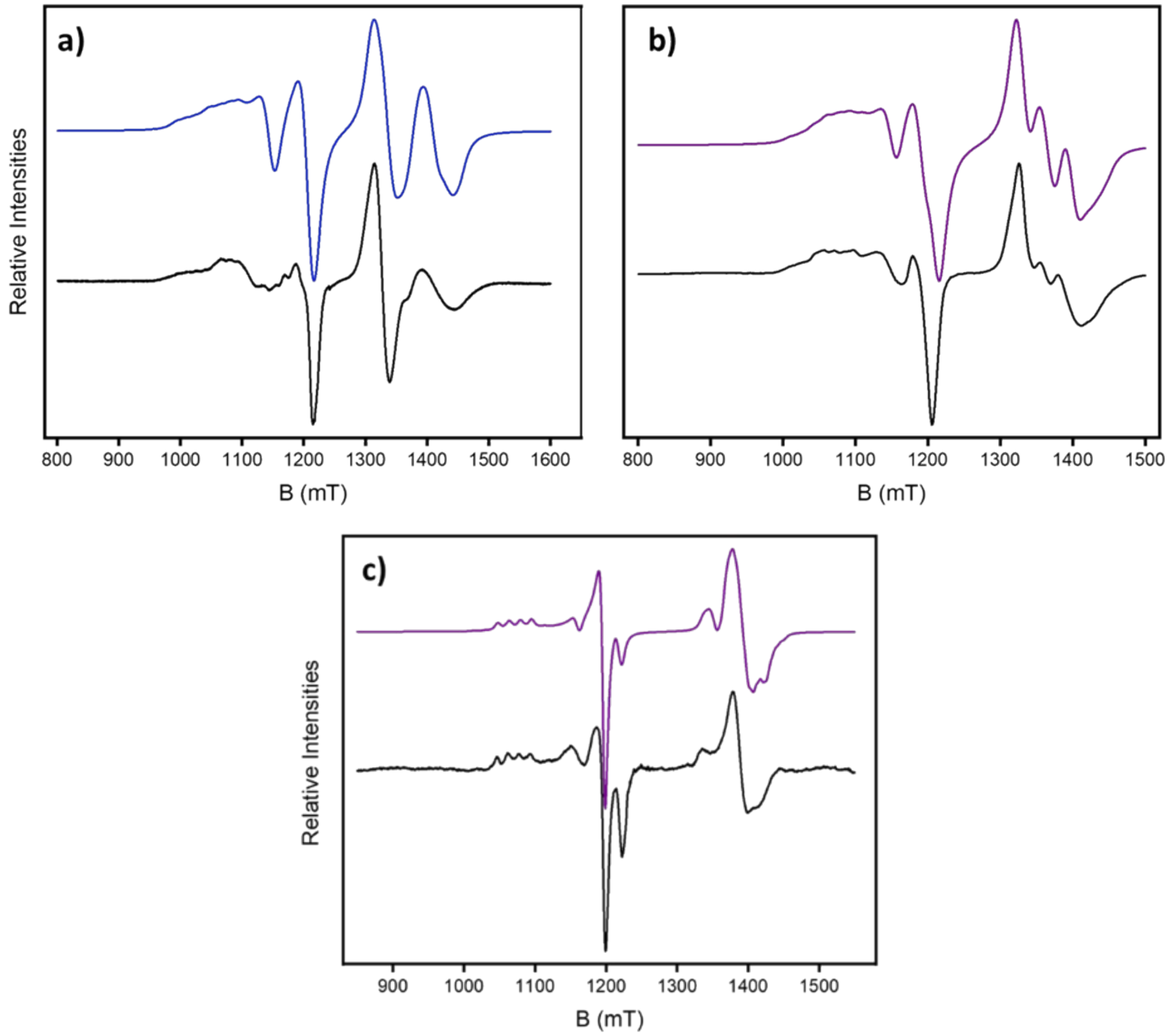

stated otherwise.

Fig. S2 CW Q-Band Powder EPR for 2 and 4, and solution for 4 CW Q-Band (ca. 34 GHz) EPR powder spectra for a) **2** at 5K (black) and simulation (blue), b) **4** at 5K (black) and simulation (purple) and c) solution spectrum for **4**

at 5K (black) and simulation (blue). Experimental frequencies: a) 34.074126 GHz, b) 34.117915 GHz and c) 34.123413 GHz.

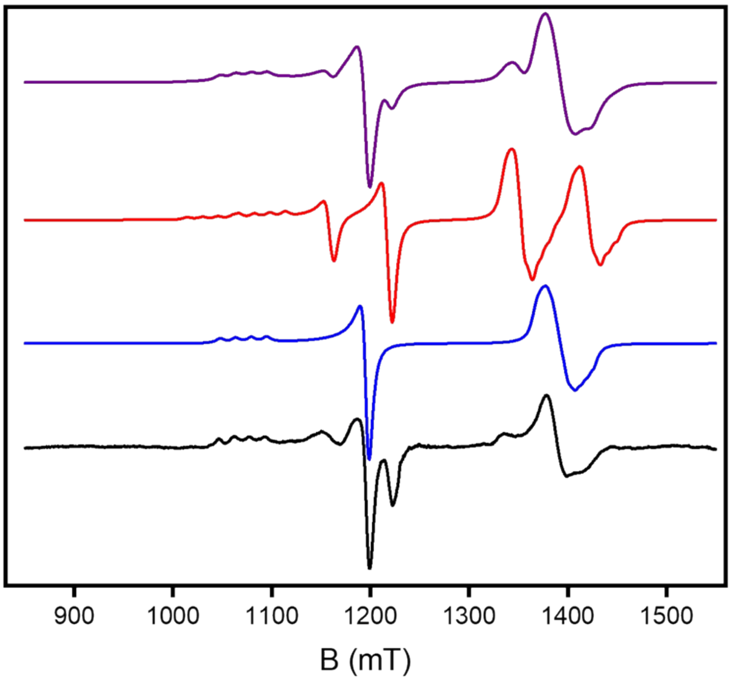

Fig. S3 CW Q-Band J coupling simulations for 4 CW Q-Band (ca. 34 GHz) EPR experimental trace (black line), simulation with 2J = 0 cm^{-1} (blue), simulation with J = 0.056 cm^{-1} (red) and combined 2J simulations (purple line).

Continuous wave EPR details and spectra for 10:

The CW EPR spectra of **10** show two isolated components, with well resolved features. As **10** has the longest separation of the Cu and {Cr_7Ni} components, the CW EPR spectra appears as a simple superposition of the independent spectra of the components (Figure S4). Simulations[S5] using a 2J = 0 can reproduce the resolution and sharpness expected for two components that do not overlap or have any exchange interaction between them. For a 1:1 3 mM solution of DCM:Toluene at 5K a best fit was found giving g-values: $g_{x,y,z}$(Cu) = 2.052, 2.045, 2.302, and $g_{x,y,z}$(Cr_7Ni) = 1.777, 1.765, 1.730, where x, y, z refer to the local g-frames of the two components. The unique z components are perpendicular to one another; the z component of the ring runs parallel through the plane of the ring.[S6] The elongated (unique axis) of the Cu^{II} ion are a set of O…Cu…O bonds that are perpendicular to the N…Cu…N bonds between the thread and the Cu^{II} ion, which are parallel to the z component of the ring. A Euler rotation of 0,90,0 (using the conventional zyz system) would be required to align the g_z

components. There is good resolution of a well-defined quartet from the ^{63,65}Cu hyperfine (I = 3/2) interaction on the g_z(Cu) component, with A_z = 450 MHz. A 2% g-Strain was applied to the {Cr_7Ni} components.

The powder sample for **10** (Fig. S4) shows the same features as per the solution samples, but with better resolution of the {Cr_7Ni} g_z component. This shows extra flexing of the {Cr_7Ni} ring for **10** due to an additional CH_2 group between the stoppers, with the resolution averaged out in the solution spectra. A best fit of the simulation for the powder sample came by initial using the parameters of the solution sample, with a small adjustment to give the optimal g values: $g_{x,y,z}$(Cu) = 2.060, 2.052, 2.310, and $g_{x,y,z}$(Cr_7Ni) = 1.787, 1.775, 1.738.

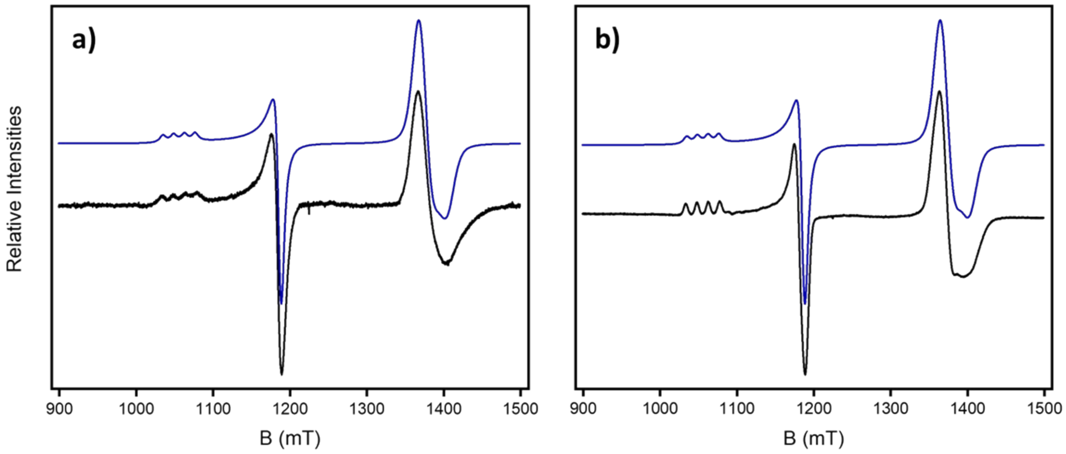

Fig. S4 CW Q-Band EPR spectra for 10 CW Q-Band (ca. 34 GHz) EPR experimental trace (black line), simulation (blue). a) Solution 5K. b) Powder 5K. Experimental frequencies: a) 34.016450 GHz and b) 34.110866 GHz.

Echo Detected Field sweep for 2:

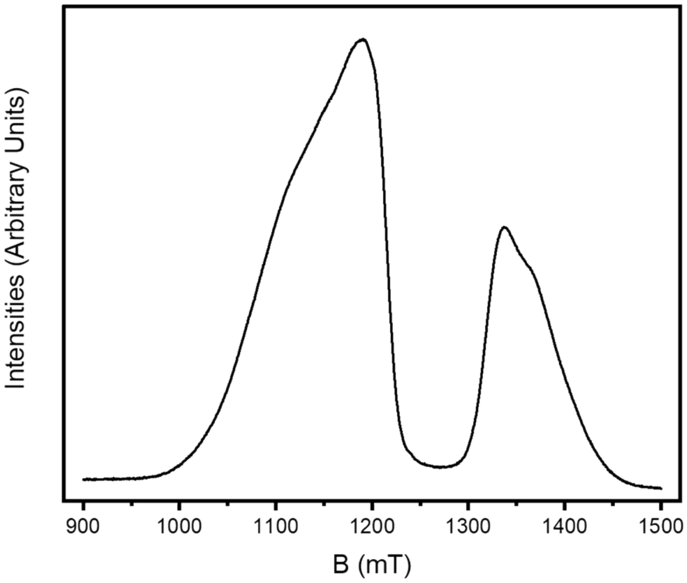

Fig. S5 Echo detected field sweep for 2 Q-Band (ca. 34 GHz) EDFS spectrum of **2** in a frozen solution (toluene, 0.2 mM) at 3K. The maxima of the Cu^{II} ion and $\{Cr_7Ni\}$ ring components are at B_0 = 1190 mT and B_0 = 1337 mT, respectively.

Pulse EPR relaxation measurements:

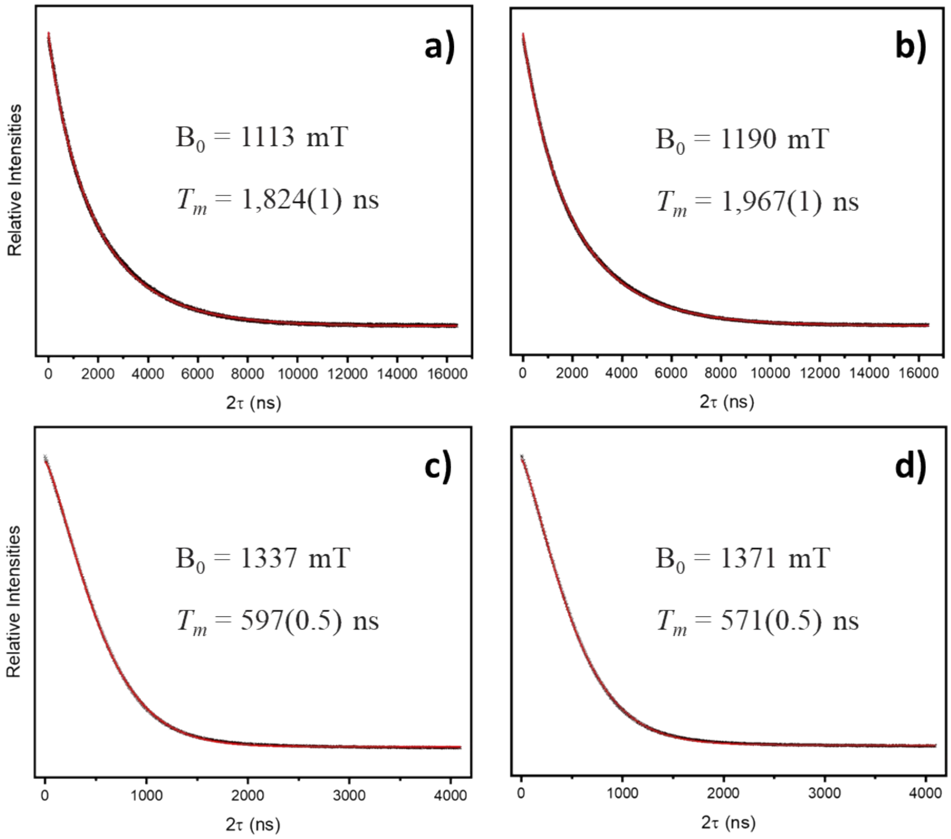

Fig. S6 Phase memory times (T_m) for 2 observed at a) 1113 mT (Cu^{II} g_z); b) 1190 mT (Cu^{II} $g_{x,y}$); c); 1337 mT ({Cr_7Ni} peak); d) 1371 mT ({Cr_7Ni} shoulder). All T_m measurements were performed with a sample concentration of 0.2 mM at 3 K. A π/2-τ-π-τ-echo sequence was used with 40 (π/2) and 80 (π) ns pulses with a tau of 300 ns. The echo decays were fit to an exponential decay with the form $I(2\tau) = I(2\tau_0)\exp(-2\tau/T_m)$.

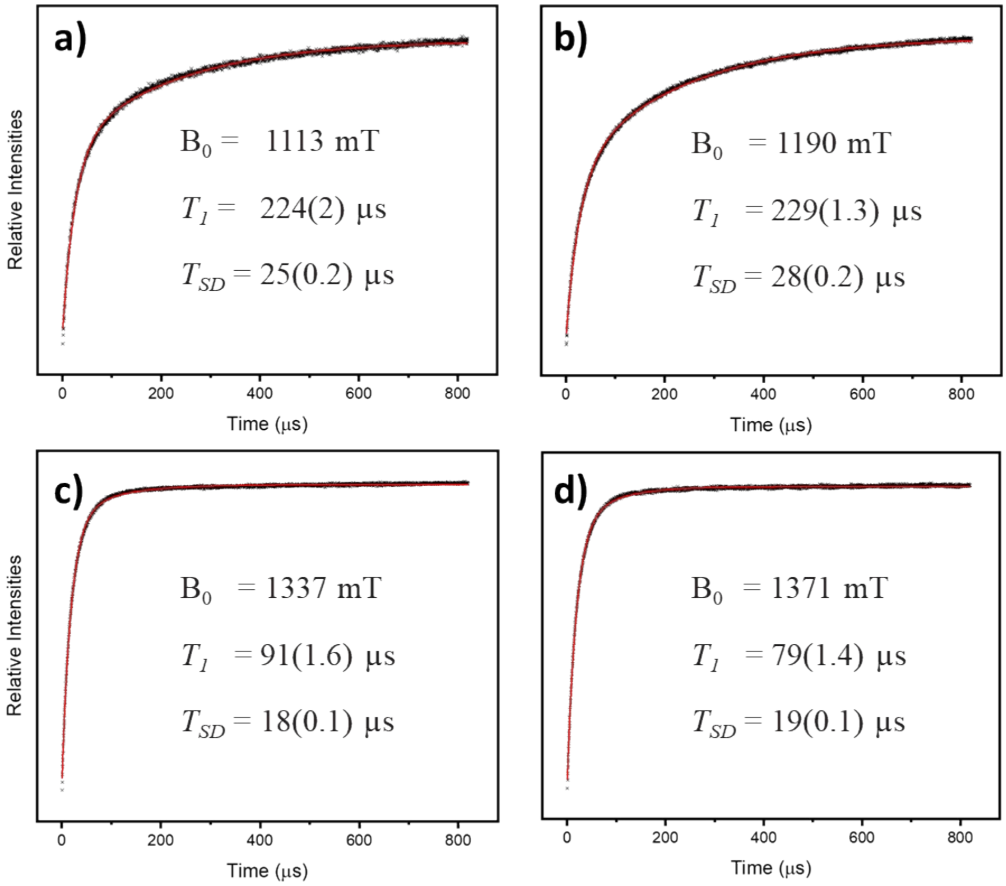

Fig. S7 Spin lattice relaxation times (T_1) for 2 observed at a) 1113 mT (Cu^{II} g_z); b) 1190 mT (Cu^{II} $g_{x,y}$); c); 1337 mT ({Cr_7Ni} peak); d) 1371 mT ({Cr_7Ni} shoulder). All T_1 measurements were performed with a sample concentration of 0.2 mM at 3 K. A π-T-π/2-τ-π-τ-echo sequence was used with; 40 (π/2), 80 (π) ns pulses with a τ of 1000 ns, for a) and b), and; 16 (π/2), 32 (π) ns pulses with a τ of 600 ns, for c) and d), . The echo decays were fit to an exponential decay with the form $I(\tau) = I_1(\tau_0)\exp(-\tau/T_1)+I_2(\tau_0)\exp(-\tau/T_{SD})$.

Details on the simulation of quantum gates

Effective Hamiltonian

We consider a system described by spin Hamiltonian (1), with a static field applied along z. We neglect here the Zeeman interaction for the nuclear spin I_2, as well as additional quadrupole terms $p(I_2^z)^2$, which do not qualitatively alter our scheme and could be probed by targeted NMR measurements.

We restrict our attention to the subspace spanned by the state vectors $|m_{S_1} = \pm 1/2, m_{I_2} = \pm 1/2\rangle$. In the limit $|g_2^z - g_1^z|\mu_B B \gg J_x, A_x$ the electronic Cu spin S_2 can be eliminated and the effective Hamiltonian in such subspace is given by

$$H = \frac{\Gamma}{2}(|1/2, -1/2\rangle\langle -1/2, 1/2| + |-1/2, 1/2\rangle\langle 1/2, -1/2|) + \lambda_1 S_1^z + \lambda_2 I_2^z \#(S1)$$

$$\Gamma = -J_x A_x \left[\frac{1}{g_2^z \mu_B B} + \frac{1}{(g_2^z - g_1^z)\mu_B B - A/2}\right]$$

This interaction is ineffective as long as $|\lambda_1 - \lambda_2| \gg \Gamma$.

However, if we apply an oscillating field ($B_1 \cos \omega t$ with $\hbar\omega = |\lambda_1 - \lambda_2|$) parallel to z, this will practically couple only to S_1^z and not to I_2^z (due to the very different magnetic moments), thus re-normalizing $\lambda_1 - \lambda_2$S7 and inducing coherent oscillations between states $|m_{S_1} = +1/2, m_{I_2} = -1/2\rangle$ and $|m_{S_1} = -1/2, m_{I_2} = 1/2\rangle$. The quarter-period of these oscillations (i.e. the time required to induce an iSWAP gate) is approximately

$$\tau = \frac{2\pi\hbar\Delta}{\Gamma g_1^z \mu_B B_1}$$

with $\Delta \approx g_1^z \mu_B B + A/2$ the energy gap between states $|m_{S_1} = +1/2, m_{I_2} = -1/2\rangle$ and $|m_{S_1} = -1/2, m_{I_2} = 1/2\rangle$. By keeping only leading contributions we obtain

$$\tau \approx \frac{4\pi\hbar B}{JAB_1}(g_2^z - g_1^z)\mu_B B \propto \frac{B^2}{JB_1}$$

which is consistent with the result of our simulations reported in Fig. S9.

Transfer of information via iSWAP

The time evolution induced by the effective Hamiltonian (S1) for a time τ corresponds to an iSWAP gate, which brings $|m_{S_1} = \pm 1/2, m_{I_2} = \mp 1/2\rangle$ to $i|m_{S_1} = \mp 1/2, m_{I_2} = \pm 1/2\rangle$. Given a generic superposition of the four states of the computational basis, this is an entangling operation. However, our initial state for information transfer is always reduced to a superposition of two states, i.e. a generic superposition of the processor with the memory in $m_{I_2} = -1/2$ (

$\frac{\alpha|1/2\rangle_1 + \beta|-1/2\rangle_1}{\sqrt{2}} \otimes |-1/2\rangle_2$) or vice-versa. Within this subspace, the phase i reduces to a single-qubit R_z rotation and hence does not create any entanglement between memory and processor.

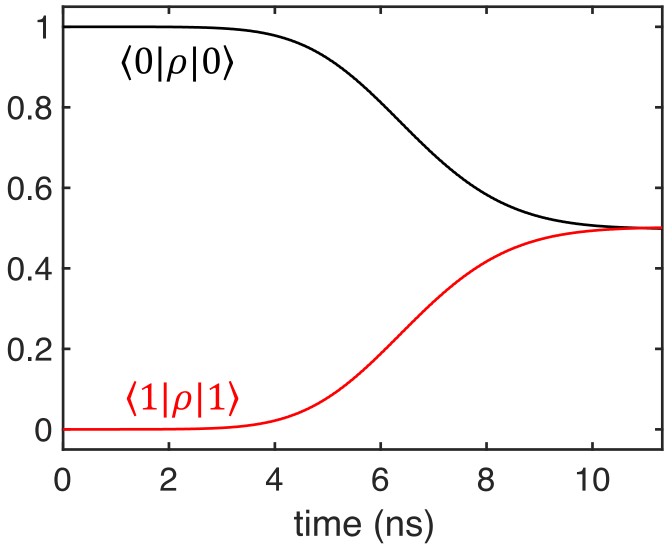

Fig. S8 Simulation of the Hadamard gate on the processing unit by a Gaussian microwave pulse of peak amplitude 5 mT, resonant with the $0-1$ transition of the proper duration. The final fidelity is 99.9 %, using a coherence time $T_M^p = 3\ \mu$s for the Cr_7Ni ring.

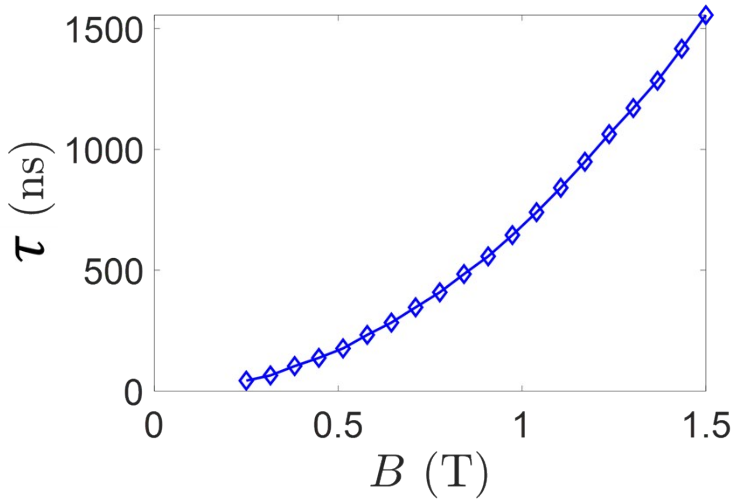

Fig. S9 Duration of the iSWAP. Time required to implement an iSWAP gate as a function of the static field. The simulated points fallow a quadratic behavior, in agreement with second order perturbation theory.

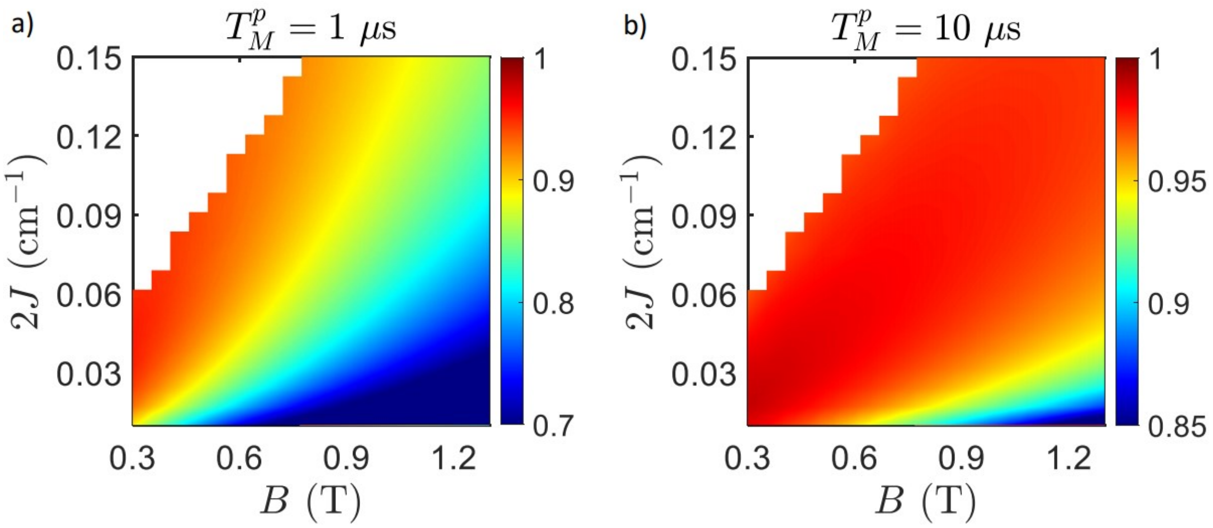

Fig. S10 Simulation of iSWAP. Fidelity as a function of external field and Cu-Cr_7Ni exchange interaction, for a) $T_M^p = 1\ \mu$s and b) $T_M^p = 10\ \mu$s. Points with low factorization (<0.9) between Cr_7Ni and Cu states have been excluded.

References:

S1. G. F. S. Whitehead, B. Cross, L. Carthy, V. A. Milway, R. Harapriya, A. Fernandez, S. L. Heath, C. A. Muryn, R. P. Pritchard, S. J. Teat, G. A. Timco, R. E. P. Winpenny, *Chem. Commun.,* 2013, **49**, 7195– 7197.

S2. S. J. Lockyer, A. J. Fielding, G. F. S Whitehead, G. A. Timco, R. E. P. Winpenny, E. J. L. McInnes, *J. Am. Chem. Soc*., 2019, **141**, 14633– 14642.

S3. (a) G. M. Sheldrick, *SADABS*, empirical absorption correction program based upon the method of Blessing. (b) L. Krause, R. Herbst-Irmer. G. M. Sheldrick, D. Stalke, *J. Appl. Cryst.,* 2015, **48**. (c) R. H. Blessing, *Acta Crystallogr.,* 1995, **A51**, 33-38.

S4. (a) G. M. Sheldrick. Acta Crystallogr., 2015, **C71**, 3-8; b) O. V. Dolomanov, L. J. Bourhis, R. J. Gildea, J. A. K. Howard, H. Puschmann, *J. Appl. Cryst*., 2009, **42**, 339–341.

S5. S. Stoll, A. Schweiger, *J. Magn. Reson*., 2006, **178**, 42.

S6. S. Piligkos, H. Weihe, E. Bill, F. Neese, H. El Mkami, G. M. Smith, D. Collison, G. Rajaraman, G. A. Timco, R. E. P. Winpenny, E.J. L. McInnes, *Chem. Eur. J.*, 2009, **15**, 3152– 3167.

S7. M. Kälin, M. Fedin, I. Gromov, A. Schweiger, A. Multiple-photon transitions in EPR spectroscopy, Chapter in Lecture Notes in Physics, 2006, DOI: 10.1007/3-540-32627-8_6.
